# Plant-Associated Representatives of the *Bacillus cereus* Group Are a Rich Source of Antimicrobial Compounds

**DOI:** 10.3390/microorganisms11112677

**Published:** 2023-10-31

**Authors:** Joachim Vater, Le Thi Thanh Tam, Jennifer Jähne, Stefanie Herfort, Christian Blumenscheit, Andy Schneider, Pham Thi Luong, Le Thi Phuong Thao, Jochen Blom, Silke R. Klee, Thomas Schweder, Peter Lasch, Rainer Borriss

**Affiliations:** 1 Proteomics and Spectroscopy Unit (ZBS6), Center for Biological Threats and Special Pathogens, Robert Koch Institute, 13353 Berlin, Germany; vatermj@web.de (J.V.); jennifer.jaehne@web.de (J.J.); herforts@rki.de (S.H.); blumenscheitc@rki.de (C.B.); schneidera@rki.de (A.S.); laschp@rki.de (P.L.); 2 Division of Pathology and Phyto-Immunology, Plant Protection Research Institute (PPRI), Duc Thang, Bac Tu Liem, Hanoi, Vietnam; thanhtamle10_2012@yahoo.co.uk (L.T.T.T.); luong3tb@gmail.com (P.T.L.); lethao0602@gmail.com (L.T.P.T.); 3 Bioinformatics and Systems Biology, Justus-Liebig Universität Giessen, 35392 Giessen, Germany; jochen.blom@computational.bio.uni-giessen.de; 4 Highly Pathogenic Microorganisms Unit (ZBS2), Center for Biological Threats and Special Pathogens, Robert Koch Institute, 13353 Berlin, Germany; klees@rki.de; 5 Institute of Marine Biotechnology e.V. (IMaB), 17489 Greifswald, Germany; schweder@uni-greifswald.de; 6 Pharmaceutical Biotechnology, University of Greifswald, 17489 Greifswald, Germany; 7 Institute of Biology, Humboldt University Berlin, 10115 Berlin, Germany

**Keywords:** *Bacillus cereus*, phylogenomics, DNA–DNA hybridization (ddH), biocontrol, plant growth promotion (PGP), biosynthesis gene cluster (BGC), kurstakin, thumolycin

## Abstract

Seventeen bacterial strains able to suppress plant pathogens have been isolated from healthy Vietnamese crop plants and taxonomically assigned as members of the *Bacillus cereus* group. In order to prove their potential as biocontrol agents, we perform a comprehensive analysis that included the whole-genome sequencing of selected strains and the mining for genes and gene clusters involved in the synthesis of endo- and exotoxins and secondary metabolites, such as antimicrobial peptides (AMPs). Kurstakin, thumolycin, and other AMPs were detected and characterized by different mass spectrometric methods, such as MALDI-TOF-MS and LIFT-MALDI-TOF/TOF fragment analysis. Based on their whole-genome sequences, the plant-associated isolates were assigned to the following species and subspecies: *B. cereus* subsp. *cereus* (6), *B. cereus* subsp. *bombysepticus* (5), *Bacillus tropicus* (2), and *Bacillus pacificus.* These three isolates represent novel genomospecies. Genes encoding entomopathogenic crystal and vegetative proteins were detected in *B. cereus* subsp. *bombysepticus* TK1. The in vitro assays revealed that many plant-associated isolates enhanced plant growth and suppressed plant pathogens. Our findings indicate that the plant-associated representatives of the *B. cereus* group are a rich source of putative antimicrobial compounds with potential in sustainable agriculture. However, the presence of virulence genes might restrict their application as biologicals in agriculture.

## 1. Introduction

At present, the replacement of harmful chemical pesticides by environmentally friendly biological means is a pressing need in agriculture worldwide. Microbes, such as bacteria and fungi, have been proven to be promising candidates for the development of efficient agents useful in sustainable agriculture. At present, endospore-forming *Bacillus* spp. and Gram-negative *Pseudomonas* spp. are the most used constituents of bioformulations applied in biological plant protection. The main advantage of bioformulations based on *Bacillus* endospores is their longevity, which makes their stability comparable with that of chemical fungicides [1].

During a survey of plant-beneficial bacteria as part of the microbiome of different plant-associated sites, such as the rhizosphere, the tissues of the inner root, and the attached insect larvae of Vietnamese crop plants (black pepper, coffee and orange trees, brown mustard, and tomato), a number of Gram-positive, endospore-forming bacteria, able to suppress common plant pathogens, were isolated. Based on their draft genome sequences, the isolates were taxonomically assigned as being members of the *Bacillaceae* family, representing four main taxonomic groups: *Lysinibacillus* spp., *Brevibacillus* spp., the *Bacillus subtilis* species complex, and the *Bacillus cereus* group [2]. Our further studies revealed that, in contrast to *Lysinibacillus* sp., the plant-associated *Brevibacilli* harbored a multitude of interesting antimicrobial peptides with a strong potential to suppress phytopathogenic bacteria, fungi, and nematodes [3]. The *Bacillus velezensis* isolates TL7 and S1, members of the *B. subtilis* species complex, were identified in large-scale trials as the most promising candidates for developing efficient biocontrol agents [4]. In this study, we focus on the plant-associated isolates belonging to the *B. cereus* group in order to investigate their potential for biocontrol and plant growth promotion.

The *B. cereus* group, also known as *B. cereus sensu lato* (*s.l*.), comprises a steadily increasing number of species, but is still plagued by taxonomic inconsistencies. Several phenotypic traits important for taxonomic assignment, such as the synthesis of the anthrax toxin and capsule, entomopathogenic crystal proteins, and the synthesis of emetic toxins (cereulide), are plasmid-encoded and can be lost during strain evolution [5]. Well-known members of the *B. cereus* group are the human-pathogenic *B. anthracis*, the entomopathogenic *B. thuringiensis*, and the opportunistic pathogen *B. cereus sensu stricto* (*s.s.*). The three species are able to cause human diseases with different severity [6]. They are closely related and harbor very similar genome sequences, which do not necessarily justify their delineation in different species. Traditionally, they have been discriminated due to properties mainly encoded by extrachromosomal elements.

*B. anthracis* (risk group 3) was identified in 1876 by the German physician Robert Koch as the causative agent of anthrax [7], the first disease that was linked to a microbe. Its virulence is based on the ability to form exotoxins and a capsule, which are encoded by the plasmids pXO1 and pXO2 [8].

The plasmid-encoded production of the highly toxic cereulide is restricted to rare emetic *B. cereus* strains occurring in some foods, whilst the production of the diarrheal-inducing enterotoxins (hemolysin BL, HBL; non-hemolytic enterotoxin, NHE; and cytotoxin K, CytK) is common in *B. cereus s.l.* [9]. Due to the extreme stability of the cyclic dodecadepsipeptide celeuride, which withstands current food processing techniques, their emetic *B. cereus* producer strains are of particular concern for human health [9].

*B. thuringiensis* (Bt), isolated 1901 by Ishikawa as “B. sotto” from the silkworm, and some years later as *B. thuringiensis* by Berliner from the meal moth [10], is an insect pathogen that is successfully used in agriculture as a biopesticide based on the production of diverse crystal toxins, also known as δ-endotoxins [11].

However, the *B. cereus* taxonomy solely based on the presence of virulence plasmids with a specific function becomes increasingly questionable in light of the recent phylogenomic data. The occurrence of *B. cereus* strains containing pXO1-like plasmids [12] and of crystal protein-harboring *B. thuringiensis* strains, which are phylogenetically related to *B. anthracis* [13], make the identification of these species a difficult task. Moreover, the occurrence of virulence genes in the *B. cereus s.l.* species cannot be excluded. Therefore, *B. cereus s.l*. strains with potential for use in sustainable agriculture can be a risk for public health and need to be carefully checked for their genomic content, also in the case of their taxonomic delineation suggests them as a “safe” species.

In this study, we aim to elucidate the potential of plant-associated members of the *B. cereus* species complex as biological plant protection. In the utilization of these strains as biocontrol agents, their potential due to their rich biosynthetic potential, but also the risks connected with the presence of virulence genes, need to be considered. Genome-based phylogenetic analyses revealed that most of the isolates were clustered within two subspecies of the *B. cereus s.s.* species. Interestingly, the isolate *B. cereus* TK1 harbored genes encoding two different crystal proteins and one vegetative insectopathogenic toxin (Vip3). Genome mining for biosynthetic gene clusters probably involved in the synthesis of antimicrobial peptides (AMPs) and direct mass-spectrometric investigation of the synthesized AMPs revealed that the isolates are promising candidates for use in sustainable agriculture. In this context, the lipopeptides kurstakin and thumolycin seem to be of special importance. A special highlight of our research is resolving the primary structure of the plasmid-encoded thumolycin pentapeptide by LIFT-MALDI-TOF/TOF fragment analysis. The inhibiting action of the *B. cereus s.l.* isolates against plant pathogens was corroborated in direct assays performed with pathogenic oomycetes, fungi, and nematodes. Finally, the plant-growth-promoting activity of some of the isolates is demonstrated. Regardless of these promising results, we have to consider the risk for public health when the *B. cereus s.l*. isolates are applied as biological means in agriculture.

## 2. Materials and Methods

### 2.1. Strain Isolation and Cultivation

Isolation from Vietnamese healthy crop plants, and insects attached on plant surfaces (Table 1), and the purification of the strains were performed as described previously [2,4]. In order to exclude vegetative cells, the samples were heat-treated at 80 °C for 20 min. Only isolates able to suppress fungal plant pathogens were selected for further characterization [2]. The cultivation of the bacterial strains and DNA isolation have been previously described [14]. The *B. cereus* group strains were cultivated on Cereus Ident agar and Cereus-selective agar, as described in Section 2.4.

### 2.2. Reconstruction of the Complete Genomes

The genome sequences of *Bacillus cereus* A22, *B. cereus* A24, *B. cereus* HD1.4B, and *B. cereus* HD2.4 were reconstructed using a combined approach of two sequencing technologies that generated short paired-end reads and long reads. The resulting sequences were then used for hybrid assembly. Short-read sequencing has been previously described [14]. Long-read sequencing was conducted in house with the Oxford Nanopore MinION with the flowcell (R9.4.1), as described previously [3]. The quality of assemblies was assessed by determining the ratio of falsely trimmed proteins by using Ideel (https://github.com/phiweger/ideel, accessed on 1 November 2021). The genome coverage of the obtained contigs was 50× in average. Genome annotation and visualization was performed as described previously [3].

### 2.3. Screening of the Virulence Genes

The screening of virulence genes in whole-genome shotgun sequences (WGS) and complete genomes was performed by using a combined analysis of the PATRIC annotation system [15] and tblastN in the 17 genomes. The most characteristic genes from *B. anthracis* Vollum, including four genes of the pXO1 plasmid (*cya*, *lef*, *pag*A, and *rep*X) and six genes of the pXO2 plasmid (*cap*A, *cap*B, *cap*C, *cap*D, *cap*E, and *rep*S), were used as the reference sequences. The tblastN threshold for both similarity and coverage was >30%, and all BLAST results were cross-checked against the PATRIC annotation, available at the Bacterial and Viral Bioinformatics Resource Center, BV-BRC, https://www.bv-brc.org/, accessed on 1 November 2021.

**Table 1:** The plant-associated *B. cereus* group isolates and their collection sites. Two *B. cereus* genomosubspecies, A, and B, were distinguished (Figure 1). The crop plants used for isolating the strains were black pepper (*Piper nigrum*) trees, tomato (*Lycopersicon esculentum*) plants, orange (*Citrus sinensis)* trees, maize (*Zea mays*), and brown mustard (*Brassica juncea*). Samples obtained from the inner root tissues were obtained after the sterilization of the root surface [2].

Accession	Size	G + C	Genes		Sample	GTDB	Collection			
	bp	%	Coding	RNA	Name	Genomospecies	Plant	Organ	Site	Date
CP085501.1	5,268,018	35.7	5533	149	A24	*B. cereus* ssp. *A*	black pepper	root	Vietnam	24 May 2018
CP085506.1	5,183,312	34.9	5450	154	HD2.4B	*B. cereus* ssp. *A*	tomato plants	rhizosphere	Vietnam, Hoai Duc, Ha Noi	23 April 2019
CP085510.1	5,183,870	34.9	5450	157	HD1.4B	*B. cereus* ssp. *A*	tomato plant	rhizosphere	Vietnam, Hoai Duc, Ha Noi	23 April 2019
JABSVB000000000.1	5,796,358	34.8	5664	80	HB3.1	*B. cereus* ssp. *A*	orange tree	rhizosphere	Vietnam, Cao Phong, Hoa Binh	17 April 2019
VDDR00000000.1	5,071,716	35.4	5619	63	A8	*B. cereus* ssp. *A*	coffee tree	root	Vietnam	9 May 2018
VEPT00000000.1	5,319,678	35.3	5332	63	A31	*B. cereus* ssp. *A*	black pepper	root	Vietnam	22 May 2018
VEPS00000000.2	6,195,299	34.7	6254	78	TK1	*B. cereus* ssp. *B*	black pepper	rhizosphere	Vietnam	9 May 2018
CP085498.1	5,310,791	35.2	5640	143	A22	*B. cereus* ssp. *B*	coffee tree	root	Vietnam	9 May 2018
JABSVF000000000.1	5,869,336	34.8	5640	80	M2.1B	*B. cereus* ssp. *B*	maize	rhizosphere	Vietnam, Phu An, Thanh Da	6 December 2019
VEPQ00000000.1	5,604,011	35.6	5466	63	A42	*B. cereus* ssp. *B*	black pepper	root	Vietnam	12 May 2018
VEPR00000000.1	5,594,617	35.6	5480	67	SN4-3	*B. cereus* ssp. *B*	maize	dead insect	Vietnam	28 May 2018
VEPV00000000.1	5,335,513	35.2	5339	58	SN1	*B. tropicus* ssp. *B*	*Ostrinia nubilalis*		Vietnam	28 May 2018
VEPW00000000.1	5,958,606	35.8	5843	93	CD3-2	*B. tropicus* ssp.	brown mustard	rhizosphere	Vietnam	28 May 2018
VEPU00000000.1	5,443,801	35.2	5389	72	SN4.1	*B. pacificus* ssp. *B*	*Ostrinia nubilalis*		Vietnam	28 May 2018
JABSVD000000000.1	5,695,940	35.1	5534	87	HD1.3	*Bacillus* sp.	tomato plant	rhizosphere	Vietnam, Hoai Duc, Ha Noi	23 April 2019
VEPX00000000.1	5,695,940	35.3	5136	65	CD3-5	*Bacillus* sp.	brown mustard	rhizosphere	Vietnam	28 May 2018
VEPY00000000.1	5,150,560	35.2	5175	61	CD3-1a	*Bacillus* sp.	brown mustard	rhizosphere	Vietnam	28 May 2018

The criteria for the presence of virulence plasmids were established as described by Liu et al. [16]. Sequences representing the different types of δ-endotoxins (Supplementary Table S1) were extracted from the NCBI data bank. Searches for the presence of genes encoding crystal proteins and toxins in the 17 plant-associated *B. cereus* genome sequences were performed with tblastN using the respective protein sequences as query.

### 2.4. Genotypic and Phenotypic Characterization of the Isolate B. cereus CD3-1a

In addition to the *B. anthracis* virulence genes mentioned above, the genome of strain *B. cereus* CD3-1a was also screened for presence of the four *B. anthracis*-specific prophage regions (dhp) described by Radnedge et al. [17]. These in silico analyses were complemented by real-time PCR assays targeting *pag*A, *cap*B, *rpo*B, and dhp61.183. Colony morphology was examined on Columbia blood agar, blood trimethoprim agar, Cereus Ident agar, and Cereus-selective agar [18].

### 2.5. Taxonomical Phylogeny Assessment

Species and subspecies delineation were performed using the Type (Strain) Genome Server (TYGS) platform [4]. Information on nomenclature was provided by the List of Prokaryotic names with Standing in Nomenclature (LPSN), available at https://lpsn.dsmz.de (accessed on 1 November 2021) [19]. The EDGAR3.0 pipeline [20] was used for elucidating taxonomic relationships as described previously [4].

### 2.6. Genome Mining

The in silico prediction of gene clusters involved in secondary metabolite synthesis was performed using the antiSMASH pipeline version 6 [21], the bioinformatic tool described by Bachmann and Ravel [22], and BAGEL4 [23].

### 2.7. Sample Preparation and Mass-Spectrometric Detection of the Bioactive Peptides

The bioactive compounds of the investigated *B. cereus s.l.* strains were detected and identified by MALDI-TOF MS, as outlined previously [24,25]. A Bruker Autoflex Speed TOF/TOF mass spectrometer (Bruker Daltonics; Bremen, Germany) was used with Smartbeam laser technology applying a 1 kHz frequency-triple Nd-YAG laser (λ_ex_ = 355 nm). Samples (2 µL) of the colony surface extracts and culture supernatants were mixed with a 2 µL matrix solution (a saturated solution of α-hydroxy-cinnamic acid in 50% aqueous ACN containing 0.1% TFA) spotted on the target, air-dried, and measured. Mass spectra were obtained by positive-ion detection in the reflector mode. The monoisotopic masses were observed. Parent ions were detected with a resolution of 10.000. The sequence analysis of peptide products was performed by MALDI-LIFT-TOF/TOF mass spectrometry in the laser induction decay (LID) mode [26]. The product ions in the LIFT-TOF/TOF fragment spectra were obtained with a resolution of 1000.

### 2.8. Antifungal, Nematocidal, and Plant-Growth-Promoting Activity Assays

Assays for activity against plant pathogens (oomycetes, fungi, and nematodes) were performed as previously described [4]. In brief, antifungal activities were assayed by placing agar plugs containing the respective fungi onto potato dextrose agar (PDA). The test bacteria were then streaked between the plugs, and the diameter of the fungal colonies as indicative for direct growth inhibition was recorded daily.

The bioassays for nematocidal activity were performed with *Caenorhabditis elegans* N2 and *Meloidogyne* sp., as described previously [4]. In the slow killing test, the nematodes were added to an agar-solidified growth medium containing the test bacteria and incubated for a period from 3 to 5 days at 25 °C. In the liquid fast killing test, overnight cultures of the test bacteria were transferred into 12-well plates containing the nematodes in a liquid M9 medium. The mortality of nematodes was defined as the ratio of dead (non-motile) nematodes to the total number of nematodes [4].

The root-knot nematode *Meloidogyne* sp. was isolated from the roots of infested pepper plants, according to Hooper et al. [27]. Tomato plantlets were grown in pots with sterilized alluvial Red River soil under subtropical climate conditions in the local greenhouse [4]. Test bacteria and second-stage juvenile (J2) nematodes were added to the pots two weeks after transplanting. Ten weeks after infesting with the nematodes, the number of knots in tomato plants was estimated [28].

Plant growth promotion assays were performed with *Arabidopsis thaliana* seedlings, as described previously [29]. Seven-day-old seedlings were dipped into a spore suspension of the test bacteria and transferred into a square Petri dish with a half-strength Murashige–Skoog medium solidified with 1% agar. After three weeks of incubation at 22 °C and a daily photoperiod of 14 h, the fresh weight of the plants was measured.

### 2.9. Data Analysis

The data obtained from the biocontrol and plant growth promotion experiments were analyzed using a one-factorial analysis of variance (ANOVA). The mean values were calculated from the results of the replicates (n ≥ 3). The Fisher´s least significant difference (LSD) test was conducted as a post hoc test for estimating significant differences (*p*≤ 0.05) between the mean values as described previously [4].

### 2.10. Gene Bank Accession Numbers of the Complete Genome Sequences

*Bacillus cereus* A22 chromosome: CP085498.1, *Bacillus cereus* A22 plasmid P1: CP085499.1, *Bacillus cereus* A22 plasmid P2: CP085500.1, *Bacillus cereus* A24 chromosome: CP085501.1, *Bacillus cereus* A24 plasmid P1: CP085502.1, *Bacillus cereus* A24 plasmid P2: CP085503.1, *Bacillus* cereus HD1.4B chromosome: CP0855510.1, *Bacillus cereus* HD1.4B plasmid P1: CP085511.1, *Bacillus cereus* HD1.4B plasmid P2: CP085512.1, *Bacillus cereus* HD1.4B plasmid P3: CP085513.1, *Bacillus cereus* HD2.4 chromosome: CP0855506.1, *Bacillus cereus* HD2.4 plasmid P1: CP085507.1, *Bacillus cereus* HD2.4 plasmid P2: CP085508.1, *Bacillus cereus* HD2.4 plasmid P3: CP085509.1.

## 3. Results and Discussion

### 3.1. Comparative Genome Analysis of the Isolates from Vietnamese Crop Plants Representing the Bacillus cereus s.l. Complex

#### 3.1.1. Genome-Based Species and Subspecies Delineation of the Plant-Associated Isolates Belonging to the *B. cereus* Group

Seventeen of the endospore-forming bacterial strains, isolated from Vietnamese crop plants and insects attached at their surface (Table 1), were previously assigned to the *Bacillus cereus s.l.* group [2]. All isolates displayed the typical features of *B. cereus*: they developed phospholipase C and hemolytic activity when cultivated on Cereus Ident and sheep blood agar plates. The phylogenetic tree obtained from the 16S rRNA sequences supported their previous taxonomic assignment as members of the *B. cereus s.l*. group, but possessed an average branch support of only 28.5% (Supplementary Figure S1), which is not sufficient for robust species delineation.

**Figure 1:**
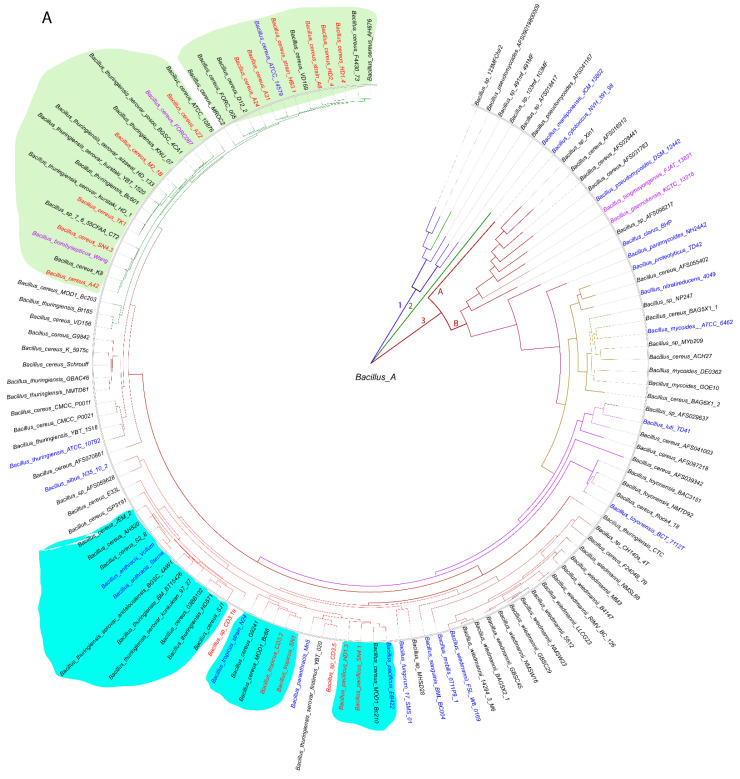

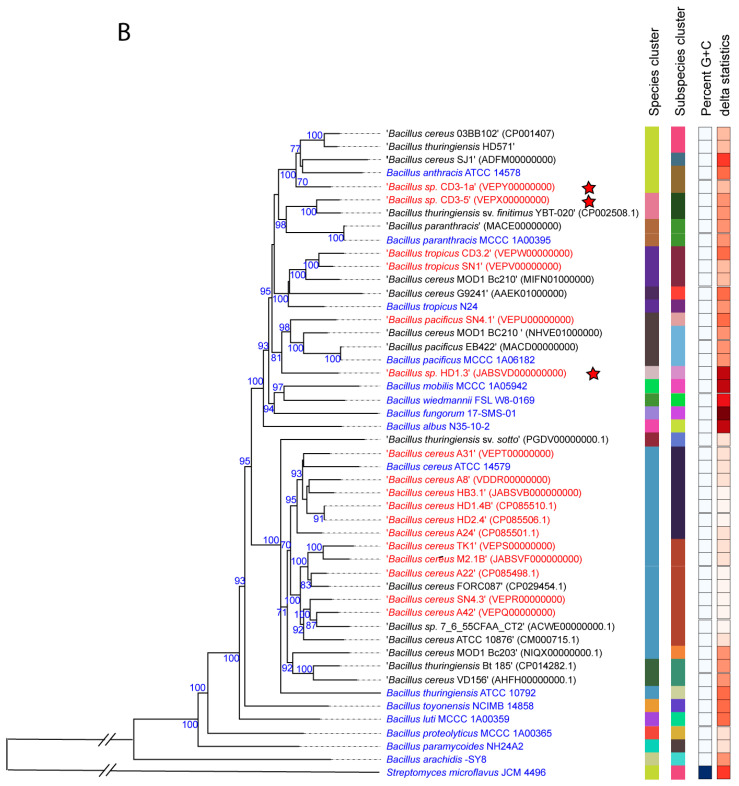

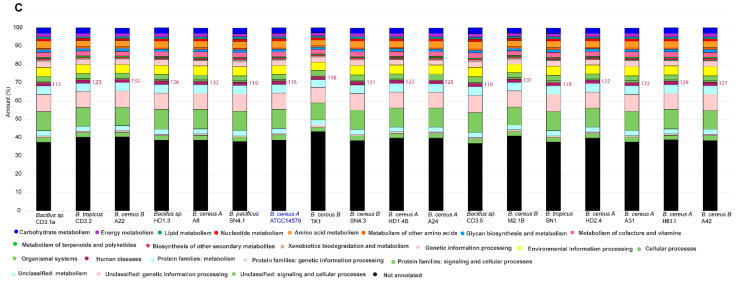
(**A**) Approximately maximum likelihood phylogenetic tree for 128 *Bacillus cereus* group genomes, calculated by EDGAR3.0 using the FastTree software (http://www.microbesonline.org/fasttree/ (accessed on 1 November 2021)). The unrooted tree was built out of a core of 1054 genes per genome. The core has 280,349 AA residues/bp per genome and 35,884,672 in total. *Bacillus*_A is a term used from GTDB (https://gtdb.ecogenomic.org (accessed on 1 November 2021)) for the genomospecies belonging to the *B. cereus* group. The tree is divided into three main branches (1–3). Cluster 3 is further divided into the subclusters 3A and 3B. The 21 type strains are labelled in blue letters. The isolates investigated in this study are indicated by red letters and all belong to the subcluster 3B. Related subclusters containing the plant-associated Vietnamese isolates are marked by irregular-colored fields. The ANI values are documented in Supplementary Figure S3. (**B**) *Bacillus cereus* tree inferred with FastMe 2.01 [30] from GBDP distances calculated from whole-proteome data using the Type (Strain) Genome Server TYGS (https://tygs.dsmz.de (accessed on 1 November 2021)). Analysis was performed using both maximum likelihood and maximum parsimony, with 16 type strains (blue letters) and 17 genome sequences obtained from the *Bacillus cereus* strains isolated from Vietnamese crop plants (red letters). In addition, 16 strains with similar proteomes obtained from the NCBI data bank were included, yielding a total of 49 proteomes. The branch lengths are scaled in terms of the GBDP distance formula *d_5_*. Putative novel genomospecies were indicated by red stars. The numbers above the branches are GBDP pseudo-bootstrap support values > 60% from the replications, with an average branch support of 87.5%. The first two colored columns to the right of each name refer to the genome-based species and subspecies clusters, specified by dDDH cutoff values of 70% and 79%, respectively. (**C**) Functional KEGG category analysis of plant-associated *B. cereus* group isolates. The type strain *B. cereus* ATCC 14,579 was included in the analysis. The number of genes associated with human diseases is indicated. A total of 125,216 KEGG functional categories (including non-annotated sequences) were found in the selected 18 contigs.

We used a whole-genome-based approach for the robust delineation of the taxonomic position of the plant-associated *B. cereus s.l.* isolates. The phylogenomic tree containing a total of 128 *B. cereus s.l.* genomes, mainly extracted from the NCBI data bank, yielded three main branches (1–3). Branch 3 was subdivided into clusters 3A and 3B. All of our isolates were distributed within the cluster 3B and were to be found related to the clusters formed by the type strains of *B. cereus*, *B. anthracis*, *B. tropicus*, and *B. pacificus* (Figure 1A).

Similar, but more detailed, results were obtained when using the Type (Strain) Genome server TYGS [31]. Our survey resulted in assigning six species and seven subspecies clusters (Figure 1B, Supplementary Figure S2). A total of 15 of the isolates were assigned to four valid species, *B. cereus* (11), *Bacillus pacificus* (1), *Bacillus tropicus* (2), and *Bacillus anthracis* (1). In the case of *B. cereus*, the dDDH values obtained after the comparison of 11 isolates with the type strain ATCC14579 exceeded the species cut off (>70%, Supplementary Table S2). At the genomic level, two subclusters were distinguished: six isolates, yielding dDDH values above the subspecies cutoff (>79%), represented the subspecies ‘A’ (*B. cereus* subsp. *cereus*), whilst five isolates showed dDDH values ranging from 72% to 74%, when compared with ATCC14579. The latter cluster formed a second subcluster ‘B’ together with *B. cereus* FORC087.1, which was clearly distinguished from subcluster ‘A’ (Supplementary Table S2). When the genomes of the members of the *B. cereus* subcluster ‘B’ were compared with the genome of *Bacillus bombysepticus* Wang [32], their dDDH values exceeded the subspecies cutoff (>79%, Supplementary Table S2). The direct comparison of FORC087 with *B. bombysepticus* Wang yielded a dDDH value of 87.8%, suggesting their close relationship. In the Genome Taxonomy Database, GTDB [33], the genomospecies *Bacillus_A bombysepticus* harbored 667 members, whilst the *B. cereus* genomospecies represented by *B. cereus* ATCC 14,579 harbored only 310 genomes (GTDB release 08-RS214, 28th April 2023).

Although *B. bombysepticus* is still not listed as a valid species in the List of Prokaryotic names with Standing in Nomenclature, LPSN [34], we propose to designate the *B. cereus* subcluster ‘B’ as genomosubspecies *B. cereus* subsp. *bombysepticus*, taking into account that the members of the ‘*bombysepticus* group’ shared dDDH values above the species cutoff with *B. cereus* ATCC 14579.

The genomes of two other isolates, SN1 and CD3-2, were assigned, according to their dDDH and Fast ANI values, to *Bacillus tropicus.* However, when compared with the *B. tropicus* type strain N24, their dDDH and ANI values were below the subspecies cutoff, indicating that these isolates form the subcluster ‘B’ together with *B. cereus* MOD1 Bc210, distinct from the *B. tropicus* type strain (Figure 1B, Supplementary Table S2).

The isolate *Bacillus* sp. CD3-1a clustered together with the *B. anthracis* type strains. However, this species delineation appeared to be questionable, since we did not detect the *B. anthracis* virulence plasmids pXO1 and pXO2 in the draft genome of CD3-1a (see next section).

Two isolates, *Bacillus* sp. CD3-5 and *Bacillus* sp. HD1.3, although distantly related to *B. tropicus* and *B. pacificus*, could not be assigned to any species present in the TYGS database (17 April 2023) and might represent novel genomospecies (Figure 1B).

#### 3.1.2. Occurrence of Virulence Genes Might Restrict the Application of *B. cereus s.l.* Isolates

The functional KEGG analysis revealed the presence of genes possibly involved in human disease in the genomes of all plant-associated *B. cereus s.l*. isolates (Figure 1C).

Within the *B. cereus* group, the occurrence of virulence factors, which are closely linked to disease symptoms [35,36], and of entomopathogenic Cry toxins [37] have been reported. In the past, these elements have been widely applied to the assignation of *B. anthracis*, *B. cereus*, and *B. thuringiensis*.

Since the genome sequence of the isolate CD3-1a formed a cluster together with the *B. anthracis* type strain ATCC 14,578 (Figure 1), we checked the CD3-1a draft genome for the presence of sequences of the characteristic anthrax toxin plasmids pXO1 and pXO2. No sequences similar to the genes encoding the Rep proteins RepX (pXO1) and RepS (pXO2) were detected in CD3-1a. Moreover, no sequences exhibiting a significant similarity with the anthrax genes of pXO1 (*cya*, *pagA*, and *lef*) and pXO2 (*capABCDE*) were found in CD3-1a and in the other plant-associated *B. cereus s.l.* isolates, excluding their taxonomic delineation as representative of the human-pathogenic *B. anthracis* species (Supplementary Table S3).

To distinguish “true” *B. anthracis* isolates from non-anthrax-causing representatives of the *B. cereus* group, a tblastN search within the genome sequence of the dhp chromosomal marker sequences, which indicate the presence of *B. anthracis*-specific prophages, was proposed [17]. None of the *B. anthracis*-specific dhp fragments could be detected in the CD3-1a genome. In addition, the real-time PCR amplification of the protective antigen *pagA* gene, the capsule *capB* gene, and the dhp61.183 gene (one of the prophage regions) using CD3-1a DNA was not achieved. A delayed amplification signal was observed for the *B. anthracis*-specific *rpoB* gene, which is known for non-anthrax strains of the *B. cereus* group [17]. In contrast to *B. anthracis*, but similar to the other isolates, CD3-1a was hemolytic when cultivated in Columbia blood agar or blood trimethoprim agar, and the genes encoding the hemolysin BL (HBL) toxin were present on the chromosome (Supplementary Table S3). The isolate also displayed phospholipase C and lecithinase activity, like the typical strains of the *B. cereus* group.

**Figure 2:**
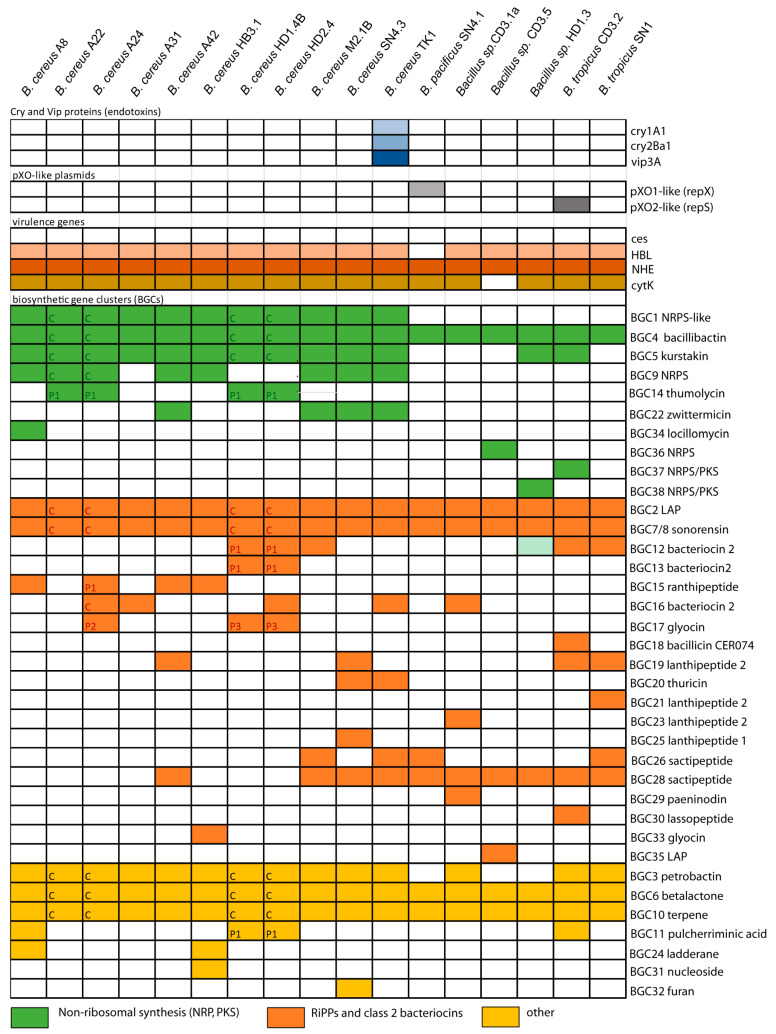
Occurrence of genes encoding entomopathogenic crystal (light blue) and vegetative (blue) proteins, the replication proteins RepS (grey) and RepX (dark grey) of *B. anthracis* pXO plasmids, and virulence genes (HBL/NHE, *cyt*K). The gene cluster for synthesizing celeuride (ces) was not detected in any of the isolates. The 36 biosynthetic gene clusters (BGCs) encoding secondary metabolites in the 17 *B. cereus s.l.* isolates were identified by AntiSMASH6.0 and BAGEL4. The location of the BGC on either the chromosome (C) or the plasmids (P1, P3) is indicated when available. Further information is presented in Supplementary Tables S3–S8.

Interestingly, the isolate *B. pacificus* SN4.1 harbored a pXO1-like *repX* gene, and the isolate *B. tropicus* CD3.2 harbored a sequence resembling the pXO2-like *repS* gene in their draft genomes (Figure 2, Supplementary Table S3), suggesting that the *rep* genes characteristic for the pOX plasmids can occur in other members of the *B. cereus s.l.* species complex. This is in line with the previous findings of Liu et al. [16].

Next, we probed the *B. cereus s.l*. isolates for the presence of other virulence genes involved in the production of toxins responsible for foodborne diseases in human beings. Cereulide, the causative agent of the emetic syndrome [9], is known to be non-ribosomally synthesized by giant peptide synthetases encoded by the *ces* gene cluster. None of our isolates harbored this gene cluster (Figure 2), suggesting that the plant-associated isolates did not represent emetic *B. cereus* strains.

By contrast, the HBL/NHE enterotoxin operons encoding the non-hemolytic enterotoxin A (NHE) and the hemolysin component BL (HBL) [38] occurred in nearly all isolates, with one exception. *B. pacificus* SN4.1 harbored the genes responsible for synthesis of NHE enterotoxin, but not the genes for hemolysin synthesis (Supplementary Table S3). HBL and NHE are the causative agents of the diarrheal syndrome in human beings, which is caused by ingestion of vegetative cells and spores that produce enterotoxins in the small intestine [39].

Due to these findings, we cannot exclude that the plant-associated *B. cereus s.l.* isolates can cause the diarrheal syndrome in human beings. The application of plant-associated *B. cereus s.l.* strains in crop protection agents represents a possible risk for public health and should be considered with care.

#### 3.1.3. Genes Encoding Insecticidal Proteins in *B. cereus* subsp. Bombysepticus TK1

Furthermore, we proved the occurrence of *cry* genes encoding entomocidal proteins (δ-endotoxins). Sequences that completely matched the crystal proteins Cry1A1 and Cry2Ba1 were detected in *B. cereus* subsp. *bombysepticus* TK1. The gene encoding the cytolytic CytK protein was detected in all *B. cereus* group isolates (Figure 2). The synthesis of δ-endotoxins is considered as a typical feature of *B. thuringiensis* [40]. However, in line with our results, Liu et al. [16] found that the ability to synthesize δ-endotoxins is widespread in different members of the *B. cereus* species complex. Thus, the presence or absence of *cry* genes cannot be considered to discriminate between the *B. cereus* and *B. thuringiensis* species.

In addition to Cry proteins, TK1 harbored a gene for the synthesis of the vegetative insecticidal protein, Vip3. Vip proteins are referred as second-generation insecticidal proteins. Vip3 proteins have insecticidal activity against Lepidopteran pests [41] and can be used for the management of various detrimental pests.

#### 3.1.4. Plasmid-Encoded Virulence Genes and Biosynthetic Gene Clusters in *B. cereus* Isolates A22, A24, HD1.4B, and HD2.4

**Figure 3:**
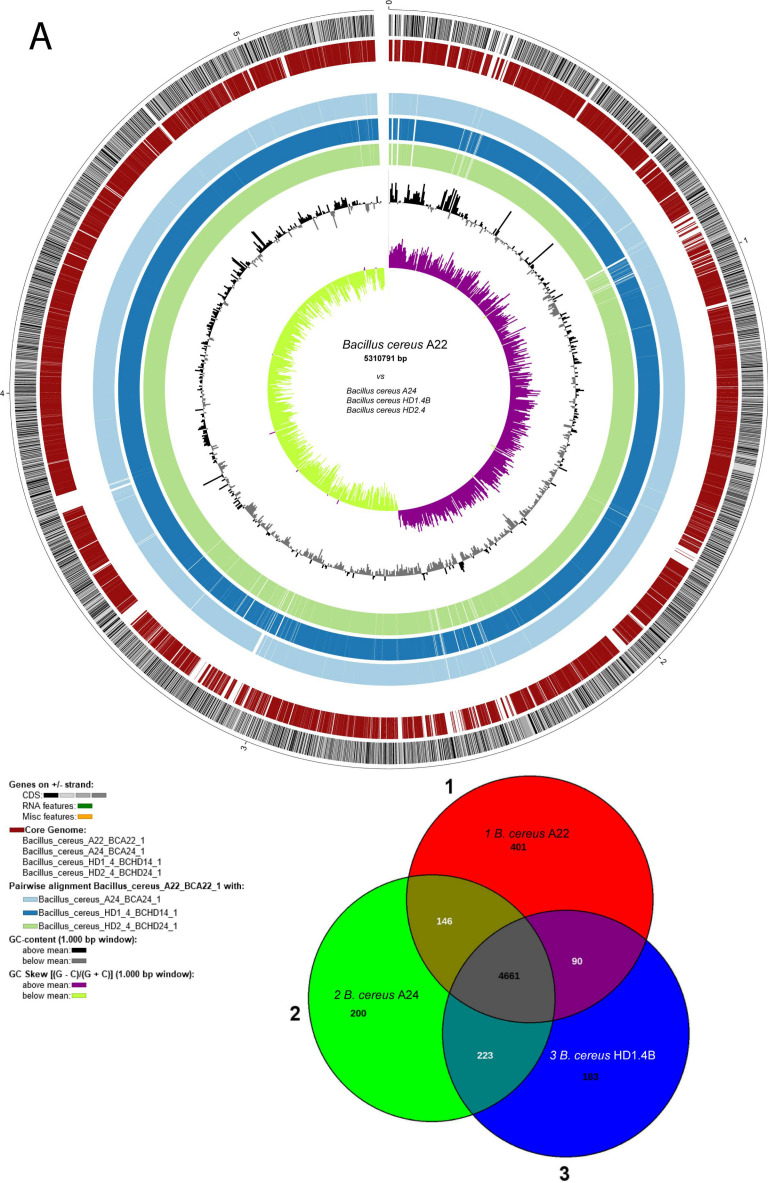

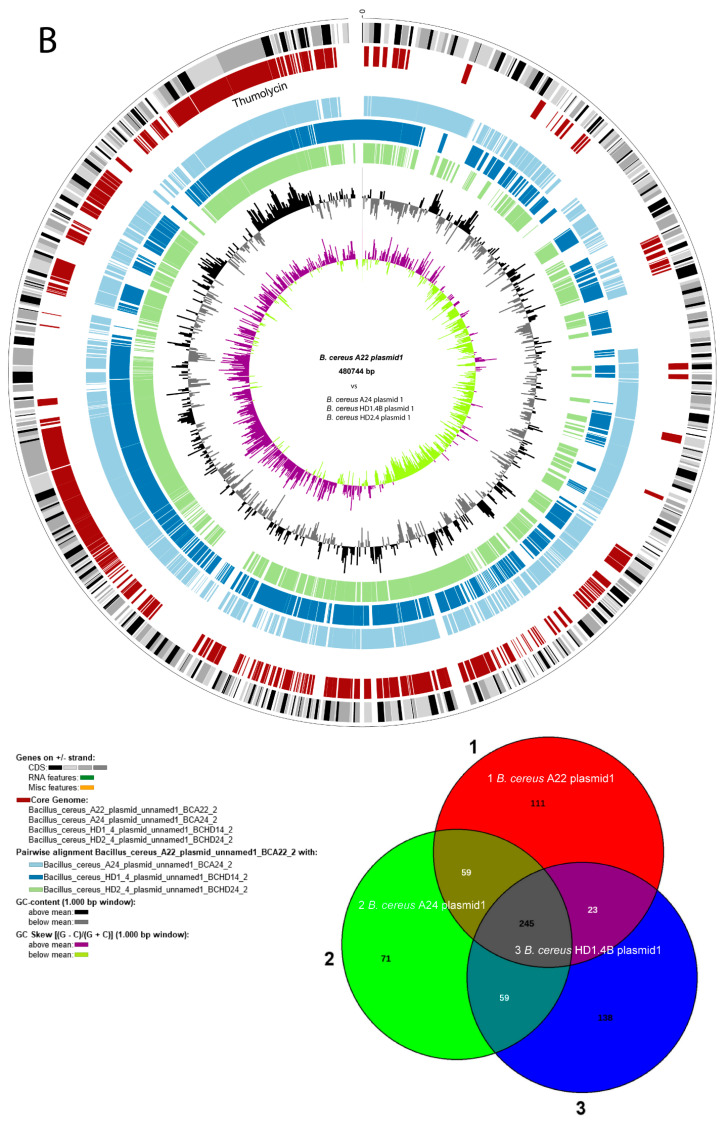
Circular plots of the *Bacillus cereus* A22 chromosome (**A**) and plasmid P1 (**B**) generated with Biocircos. The Venn diagrams below show the comparison of A22 with the chromosomes and plasmid P1 of *B. cereus* A24 and HD1.4B. From outer to inner circle: Genes (CDS) on +(1)/− strand (2); core genome, brown (3); GC content (1000 bp window) above the mean: black, below mean: grey (4); GC skew ((G-C)/(G+C)) (1000 bp window), above mean: purple, below mean: light green (5). The grey line within the inner circle shows deviations of the average GC content. The 30 kb thumolycin gene cluster is part of the core genome in all four plasmid P1 species.

A first survey of the draft genome sequences for the presence of gene clusters encoding lipopeptides revealed that *B. cereus* ssp. *bombysepticus* A22 and the *B. cereus* ssp. *cereus* strains A24, HD1.4B, and HD2.4 harbored gene clusters similar to the thumolycin gene cluster, previously detected in *B. thuringiensi* BMB171 [42]. This finding prompted us to sequence completely the four strains using the nanopore sequencing technology (see Materials and Methods). The complete genomes consisted of one single chromosomal DNA molecule and extrachromosomal DNA elements, bearing the features of plasmid DNA (Figure 3). The chromosomes of all four isolates contained more than 5000 kb. The large P1 plasmids of A22 and A24 contained 480,744 bps and 471,669 bps, respectively. The smaller P2 plasmid of A22 contained 93,778 bps. Small plasmids not exceeding 12 kb were detected in A24 (P2), HD1.4B (P3), and HD2.4 (P3). The DNA elements found in HD1.4B and HD2.4 were nearly identical, suggesting that both isolates represented clones of the same strain. Both harbored one chromosome and three plasmids of nearly identical size and gene content (Supplementary Table S4). The presence of plasmid-specific Rep proteins in all extrachromosomal elements was corroborated by using the SEED and the RAST annotation system [43] (Supplementary Figure S4).

Comparing *B. cereus* ssp. *cereus* A22 with the representatives of the *B. cereus bombysepticus* clade (*B. bombysepticus* Wang, FORC087, ATCC10876, A42, M2.1B, SN4.3, and TK1) yielded 113 singletons, including two catalases, 5-methylcytosine-specific restriction enzyme A, and other restriction enzymes. Comparing *B. cereus* ssp. *bombysepticus* A24 with the representatives of the *B. cereus cereus* clade (ATCC14579, A22, HD1.4B, HD2.4, A8, HB3.1, and A31) revealed 367 singletons, including proteins involved in conjugative transfer, the AlwI family type II restriction endonuclease, and urease subunits and associated proteins.

The potential virulence factor phosphatidylinositol-specific phospholipase C (PI-PLC), a characteristic marker of the *B. cereus* group [38], was encoded by the large P1 plasmids of A22, A24, HD1.4B, and HD2.4. PI-PLCs catalyze the cleavage of the membrane lipid phosphatidylinositol (PI), or its phosphorylated derivatives, to produce diacylglycerol (DAG) and the water-soluble head group, phosphorylated *myo*-inositol [44].

The annotation of the chromosomal elements detected in A22, A24, HD1.4B, and HD2.4 is summarized in Supplementary Table S4. Surprisingly, the plasmid P1 sequences from HD1.4B and HD2.4 harbored three genes with similarity to the NHE/HBL enterotoxin operons. These genes are known to be located on the chromosome. In fact, the chromosomes of the four isolates, including HB1.4 and HD2.4B, harbored the complete NHE/HBL gene set (Supplementary Figure S5).

Many metabolic features were found to be encoded by the large P1 plasmids harboring more than 500 coding genes. In addition to the thumolycin gene cluster, present in the large plasmids of all four isolates, two other BGCs encoding pulcheriminic acid and the bacteriocin cerein 7B precursor were found to be located in the large plasmids of HD1.4B and HD2.4.

Interestingly, in addition to the chromosomal-encoded type 1 restriction modification systems (RM) [45], type 1 RM gene clusters encoding the subunits M, S, and R were present in the 481 kb plasmid P1 of A24 and in the 94 kb plasmid P2 of A22. A fragmentary type III RM system consisting of RMIII helicase and the methylation subunit flanked by UvrD helicase and a transposase was detected in the P1 plasmid of HD2.4

A gene cluster detected in plasmid P1 of the *B. cereus* strains was similar to the anthrose BGC, previously described in *B. anthracis* Sterne [46]. The anthrose-containing oligosaccharide attached at the surface of the exosporium might contribute to enhanced survival rates under multiple stress conditions. Our results are in line with previous results of Dong et al. [47] demonstrating that the presence of anthrose-containing exosporia is not restricted to *B. anthracis*.

The complete operon for *myo*-inositol catabolism was detected in the large plasmids of all the four *B. cereus* isolates. The gene cluster was found to harbor the genes encoding the same enzymes as the *my*o-inositol operon previously detected in the chromosome of *B. subtilis* [48]. The presence of repeats and mobile elements in the flanking regions suggested that the operon might be acquired by horizontal gene transfer (Supplementary Figure S6).

### 3.2. Genome Mining for Biosynthetic Gene Clusters (BGCs) Encoding Secondary Metabolites

Antimicrobial compounds belong to structurally diverse groups of molecules, such as non-ribosomal peptides (NRPs) and polyketides (PKs), ribosomally synthesized and post-translationally modified (RiPPs) and unmodified (class 2 bacteriocins) peptides [49,50]. Genome mining using the software pipelines of antiSMASH6.0 [20], PKS/NRPS Analysis [21], and BAGEL4 [22] was performed with the genomes of all the Vietnamese isolates of the *B. cereus sensu lato* complex. The results were subsequently compared with the MIBiG database [51] in order to distinguish between characterized and uncharacterized BGCs. Our survey yielded a total of 209 BGCs representing 36 different gene clusters involved in the biosynthesis of secondary metabolites. Only a few, such as the siderophores petrobactin (BGC0000942) and bacillibactin (BGC0000309), zwittermicin (BGC0001059), locillomycin (BGC0001005), and pulcherrimic acid (BGC0002103), were listed in the MIBiG data bank. Two BGCs, kurstakin and thumolycin, although not listed in the MIBiG repository, were identified due to their similarity to genes already deposited in the NCBI data bank. Most of the BGCs exhibited no or only low similarity to the known BGCs present in the MIBiG data bank. Five BGCs encoding bacillibactin, RiPPs (2), betalactone (1), and terpene (1) were found conserved in all *B. cereus s.l.* isolates (Figure 2). An overview about the BGC species detected in the plant-associated *B. cereus* isolates is presented in Supplementary Table S5.

#### 3.2.1. Non-Ribosomally Synthesized Antimicrobial Peptides (NRPs) and Polyketides (PKs)

**Figure 4:**
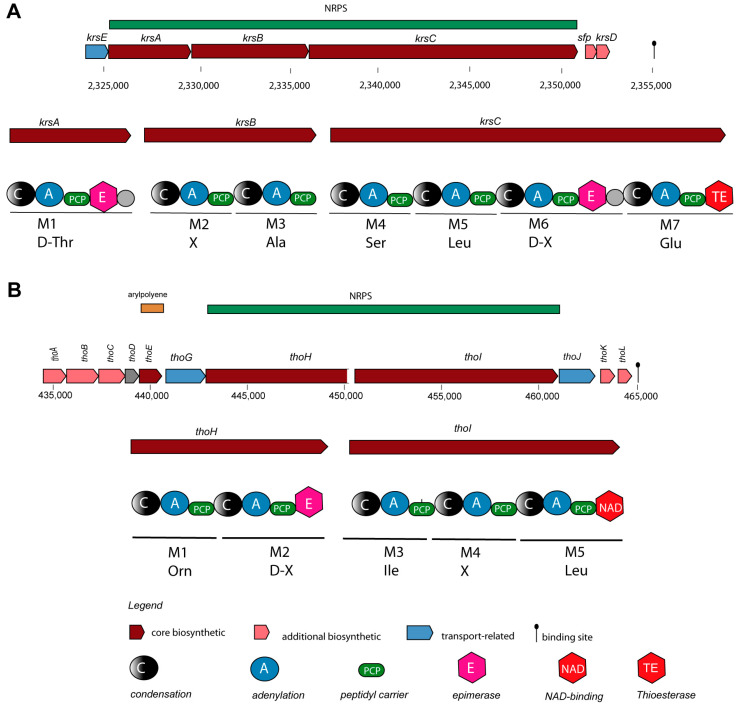
Gene cluster and domain structure involved in the non-ribosomal synthesis of cyclic lipopeptides in *B. cereus* A22. (**A**) The kurstakin (*krs*) gene cluster is located on the chromosome of A22 in the range of 2325–2355 kb. The amino acid sequence deduced from the adenylation domains was experimentally corrected and completed by LIFT-MALDI-TOF/TOF MS (see Table 2). (**B**) The thumolycin (*tho*) gene cluster resides in the *B. cereus* A22 plasmid 1 between 435 kb and 465 kb. The domain structure of ThoH and ThoI, including the amino acids deduced from their adenylation domains, is shown. The complete amino acid sequence determined by LIFT-MALDI -TOF/TOF MS is shown in Table 3. Further domains were detected in ThoC (A), ThoE (KS), ThoK (TE), and ThoL (ACPS).

NRPs are secondary metabolites that are synthesized through giant multi-modular peptide synthetases [52]. The complete *krs* gene cluster (BGC5) encoding the cyclic lipoheptapeptide kurstakin [53] was found to be widely distributed and occurs in the genomes of most *B. cereus sensu lato* isolates, except *B. pacificus* SN4.1, *B. tropicus* SN1, and *Bacillus* sp. CD3-1a and CD3.5. Kurstakin is responsible for biofilm formation [54]. Although kurstakin was present in the majority of the investigated isolates (13/17 strains), the kurstakin gene cluster, containing the genes *krsE, krsA, krsB, krsC, sfp*, and *krsD* (Figure 4A), is not listed in the MIBiG data bank.

The lipopeptide thumolycin, recently detected in *B. thuringiensis* BMB171, enabled the bacterium to develop a broad spectrum of antimicrobial and nematocidal activities [42]. Unfortunately, the structure of the lipopeptide is still not resolved. We detected the thumolycin (*tho*) gene cluster (BGC14) in plasmids of the *B. cereus* strains A22, A24, HD1.4B, and HD2.4. The genes of the *tho* cluster spanned around 30 kb. Two multimodular non-ribosomal peptide synthetases (ThoH and ThoI) synthesized a putative pentapeptide Orn-D-X-Leu/Ile-XS-Leu (Figure 4B). The *tho*C-, *tho*D-, and *tho*E-encoded proteins are probably involved in the synthesis of the fatty acid chain [42].

**Figure 5:**
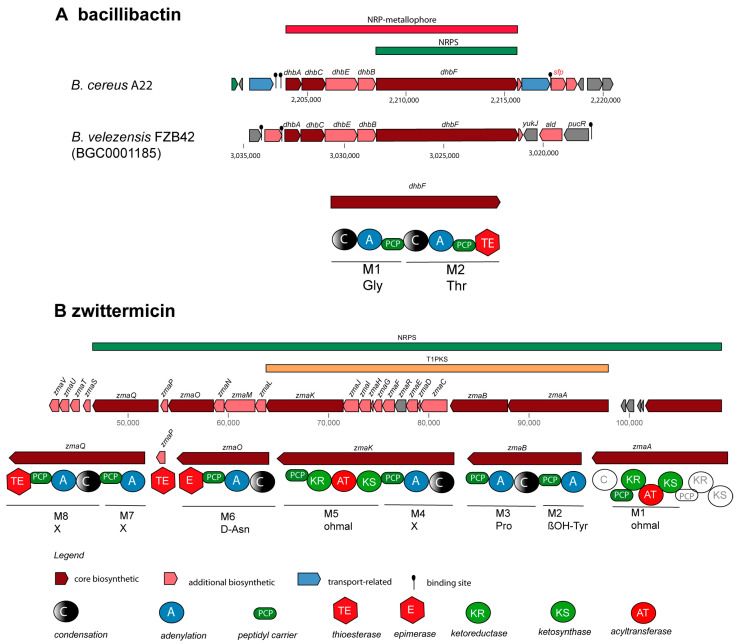
Gene clusters involved in the non-ribosomal synthesis of bacillibactin and zwittermicin. The siderophore gene cluster in the *B. cereus* A22 chromosome. (**A**) Comparison of the corresponding *B. velezensis* FZB42 gene cluster revealed the presence of the *sfp* gene downstream of the ballibactin transcription unit as a unique feature. (**B**) The gene cluster for the synthesis of the PK/NRP hybrid zwittermicin in *B. cereus* SN4-3.

Fragments of the locillomycin gene cluster [55] (BGC34) were detected in *B. cereus* A8 (Supplementary Table S5). To the best of our knowledge, to date, the locillomycin gene cluster has been detected only in members of the *B.subtilis* species complex. The gene cluster for the synthesis of the catecholic iron siderophore bacillibactin, 2,3-dihydroxybenzoyl-Gly-Thr trimeric ester, has been previously reported in the genomes of *B. subtilis* [56] (BGC0000309) and *B. velezensis* FZB42 [57] (BGC0001185). Its non-ribosomal synthesis was found to be dependent on Sfp (phosphopantetheinyl transferase) [58]. BGCs with a similar structure as that of BGC0000309 and BGC0001185 were detected in all 17 *B. cereus sensu lato* isolates investigated in this study. Whilst the core structure of the bacillibactin transcription unit was well conserved, a *sfp* gene in the flanking region was identified as a unique feature for the *B. cereus* bacillibactin operon (Figure 5A). This is in contrast to the operon structure in the *B. subtilis* species complex, where the *sfp* gene is located in a more remote location, downstream flanking the surfactin operon [58].

The gene cluster for the synthesis of the aminopolyol antibiotic zwittermicin was detected in four *B. cereus* genomes. The highly polar zwittermicin A (ZmA) possesses antiprotist and antibacterial activities, and consists of numerous ethanolamine and glycolyl moieties flanked by N-terminal D-serine and an unusual amide generated from ß-ureidoalanine. The aminopolyol structure of the final product results from different processing events of the NRPS/PK hybrid precursor molecule (Figure 5B), in which a multitude of gene products of the *zma* gene cluster are involved [59].

In addition, numerous uncharacterized NRPs, PKS, and NRP/PKS hybrids were found (Supplementary Tables S5 and S6). A unique gene in *B. tropicus* CD3.2, located downstream of an uncharacterized NRP + PK cluster (BGC37, Supplementary Figure S7), encoded a putative necrose-inducing protein (NPP1 family) [60].

#### 3.2.2. Gene Clusters Representing RiPPs and Bacteriocins

In contrast to polyketides and peptides, which are synthesized independently from ribosomes, numerous peptides with antimicrobial activity (bacteriocins) are synthesized by a ribosome-dependent mechanism. According to Zhao and Kuipers [49], several groups of ribosomally synthesized peptides (RiPPs) can be identified:

- Class I: post-translationally modified peptides smaller than 10 kDa.
- Class II: small (<10 Da), unmodified peptides with or without a leader sequence.
- Class III: peptides larger than 10 kDa.

RiPPs, such as lanthipeptides (class1 and class2), linear azol(in)e-containing peptides (LAPs), lassopeptides, sactipeptides, thiopeptides, and representatives of the class II unmodified bacteriocins, such as UviB peptides (holin-like proteins), were detected in the *B. cereus* group isolates applying the antiSMASH and BAGEL4 toolkits (Supplementary Figures S8 and S9). Many RiPP biosynthetic proteins recognize and bind their cognate precursor peptide through a domain known as the RiPP recognition element (RRE) [61]. The detection of RRE domains using antiSMASH-supported genome mining was helpful in identifying the BGCs involved in the synthesis of RiPPs, which did not contain known core peptide-encoding sequences [62].

A total of 19 different BGCs encoding RiPPs and unmodified class 2 bacteriocins were detected. Only six encoded precursor peptides with an apparent similarity to known RiPPs: BGC20 (thuricin), BGC17 and BGC18 (sublancin/CER074), BGC29 (paeninodin), and BGC26 (thurincin H) (Supplementary Tables S5 and S7).

Antimicrobial lanthipeptides (lantibiotics) are post-translationally highly modified and contain the thioether amino acid lanthionine as well as several other modified amino acids [63]. LanA precursor peptides consist of an N-terminal leader peptide and a C-terminal core region. The first step in post-translational modification is the activation and elimination of water from the Ser and Thr residues forming dehydroalanine (DhA) and dehydrobutyrine (DhB), respectively. Then, ß-thioether cross-links are generated between the DhA, DhB, and the Cys residues. The modifying enzymes involved in the formation of the thioether link in class AI lanthipeptides are the dehydratase LanB and the cyclase LanC. The modification of A2 lanthipeptides is accomplished by LanM, containing the dehydratase and the cyclase domain in one protein. Classes A3 and A4 lanthipeptides are modified by LanKC and LanL, respectively [64]. We detected a BGC encoding a representative of the A1 lanthipeptides in *B. cereus* SN4.3 (BGC25). Four genes encoding precursor peptides similar to paenibacillin and subtilomycin were identified within BGC25. BGCs encoding A2 lantibiotics similar to plantaricin (BGC19), thuricin (BGC20), lichenicidin (BGC21), salivaricin (BGC23), and paenibacillin (BGC25) occurred in several *B. cereus* isolates (Supplementary Tables S5 and S7).

Gene clusters encoding LAPS were found to be widely distributed in *B. cereus* and related species. LAPS are characterized by the post-translational modification of the precursor peptide, yielding thiazol(in)e and (methyl)oxazol(in)e heterocycles. Modifying enzymes are the FMN-dependent dehydrogenase (SagB) and cyclodehdratase (SagC and YcaO) [65]. BGC2 (Supplementary Table S7) was identified as being member of the TOMM class (thiazole/oxazole-modified microcins), characterized by a gene cluster consisting of a cyclodehydratase gene and associated genes encoding dehydrogenase and a maturation protein. The core region of the TOMM precursor leader peptide contained a region enriched with Cys residues (BGC7/8), which is typically for the hetero-cycloanthracin/sonorensin family [66].

A glyocin-encoding gene cluster (BGC17) was detected in the plasmid P2 of *B. cereus* A24. Glyocins are defined as post-translationally glycosylated RiPPs with antimicrobial activity [65,67]. BGC17 resembled sublancin, which is an S-linked glycopeptide coding a SunS family peptide S-glcosyltransferase and a bacillicin CER074 peptide (BGC0001863) containing a glucose attached to a cysteine residue [68]. A second gene cluster (BGC33) harboring genes encoding a glycosyltransferase and a putative 75 aa precursor peptide was detected in *B. cereus* HB3.1 (Supplementary Table S5).

Lassopeptides are characterized by an N-terminal macrolactam ring threaded by the C-terminal tail. A cysteine protease B and a lactam synthetase C are necessary for the post-translational modification of the precursor peptide [69]. Two gene clusters, probably encoding lassopeptides, were identified. BGC29 harbored, in addition to a structural gene (*paeA*) for the synthesis of paeninodin lasso peptide [70], the genes for the synthesis of the essential components of post-translational modification, *paeB1* (PQQD family protein), *paeB2* (cysteine protease), and *paeC* (lactam ring closing cyclase). Four copies of the lasso precursor gene and split B1 and B2 genes were detected in BGC30 (Supplementary Table S7).

Two gene clusters (BGC26 and BGC28), encoding the radical S-adenosylmethionine (rSAM) enzyme, necessary for the post-translational modification of sactipeptides, occurred in the representatives of the *B. cereus* group. A well-known representative of sactipeptides is subtilosin A (SboA), synthesized by *Bacillus subtilis*. The rSAM enzyme (AlbA) catalyzes the linkage of a thiol with an α-carbon of a functional amino acid residue [71]. BGC26 harbored genes involved in the synthesis and rSAM-dependent modification of a thurincin H-like precursor peptide. A gene encoding a protein containing an N-terminal radical SAM domain (pfam04055) and a C-terminal pfam08756 domain with a CxCxxxxC motif (BmbF) was detected in BGC28. In contrast to *B. subtilis*, the YfkA and YfkB regions, originally reported as separate ORFs in *B. subtilis*, were found fused in the *B. cereus* gene cluster (Supplementary Table S7).

Like the structurally related sactipeptides, the thioether linkage in ranthipeptides is generated via a radical-initiated mechanism. However, ranthipeptides do not contain α-carbon links and were recently designated as non-α thioether peptides [72]. The ranthipeptide gene cluster (BGC15) detected in four *B. cereus* isolates harbored a gene encoding the rSAM protein belonging to the MoaA/NifB/PqqE/SkfB superfamily (Supplementary Table S7).

A gene cluster (BGC7/8), involved in the synthesis and modification of an 82 aa precursor thiopeptide belonging to the heterocycloanthracin/sonorensin family [73], occurred in all *B. cereus* group isolates. Its C-terminal region contained an extended repeat region with Cys at every third residue (Supplementary Table S7).

Two different subclasses of bacteriocin class II peptides were detected: the holin-like BhlA encoding genes (BGC12 and BGC16) and a cluster (BGC13) harboring a gene encoding a cerein-like prepeptide belonging to the Blp family. Similar as in lanthipeptides, the Blp family prepeptides are characterized by a conserved GlyGly processing site between the N-terminal leader and the C-terminal core peptide region [74]. The BhlA holin of *Bacillus pumilus* causes bacterial death by cell membrane disruption [75]. In addition to the genes encoding leaderless BhlA peptides, the holin gene clusters harbored genes encoding muramidases (GH25 glycosyl hydrolases) that hydrolyze the peptidoglycan cell wall (Supplementary Table S7, Supplementary Figures S8 and S9).

#### 3.2.3. Other Antimicrobial Secondary Metabolites

Seven BGCs encoding other antimicrobial secondary metabolites were identified in the plant-associated *B. cereus* isolates (Supplementary Figure S7, Supplementary Table S8). Three of them, BGC3 (=BGC0000942, petrobactin), BGC11 (=BGCBGC0002103, pulcherriminic acid), and BGC32 (=BGC0000914, furan), were similar to the known BGCs deposited in the MiBIG data bank.

The *asbABCDEF* gene cluster is responsible for the biosynthesis of petrobactin, a catecholate siderophore that functions in both iron acquisition and virulence [76]. We detected the petrobactin gene cluster in the genomes of 14 isolates. Only *B. pacificus* SN4-1 and HD1-3 and *Bacillus* sp. CD3.5 did not harbor the BGC0000942 cluster, which is common in most representatives of the *B. cereus* group [77].

Pulcherriminic acid is a cyclic dipeptide able to chelate Fe^3+^ [78]. Due to its high affinity to Fe ions, *Bacillus* strains producing pulcherriminic acid compete successfully with other microorganisms in low iron environments. A gene cluster similar to the pulcherriminic acid synthesis cluster in *B. subtilis* (BGC0000914) was detected in the *B. cereus* HD1.4B and HD2.4 plasmid sequences (Supplementary Table S4) and in the draft genomes of *B. cereus* A8 and *B. tropicus* CD3-2.

Several genes of BGC32, possibly involved in the synthesis of a furan-like metabolite, showed a striking similarity to the methylenomycin A gene cluster in *Streptomyces coelicolor* [79].

Four BGCs did not show similarity to any characterized biosynthetic gene clusters. BGC6 possibly encoded ß-lactone-harbored genes with similarity to the genes flanking the plipastatin BGC in *B. subtilis*. BGC24 contained several genes of the carbohydrate metabolism, probably involved in the synthesis of ladderane. BGC10 and BGC31 encoded enzymes for the synthesis of terpene and nucleoside metabolites, respectively.

### 3.3. Detection of Bioactive Peptides by MALDI-TOF Mass Spectrometry

**Figure 6:**
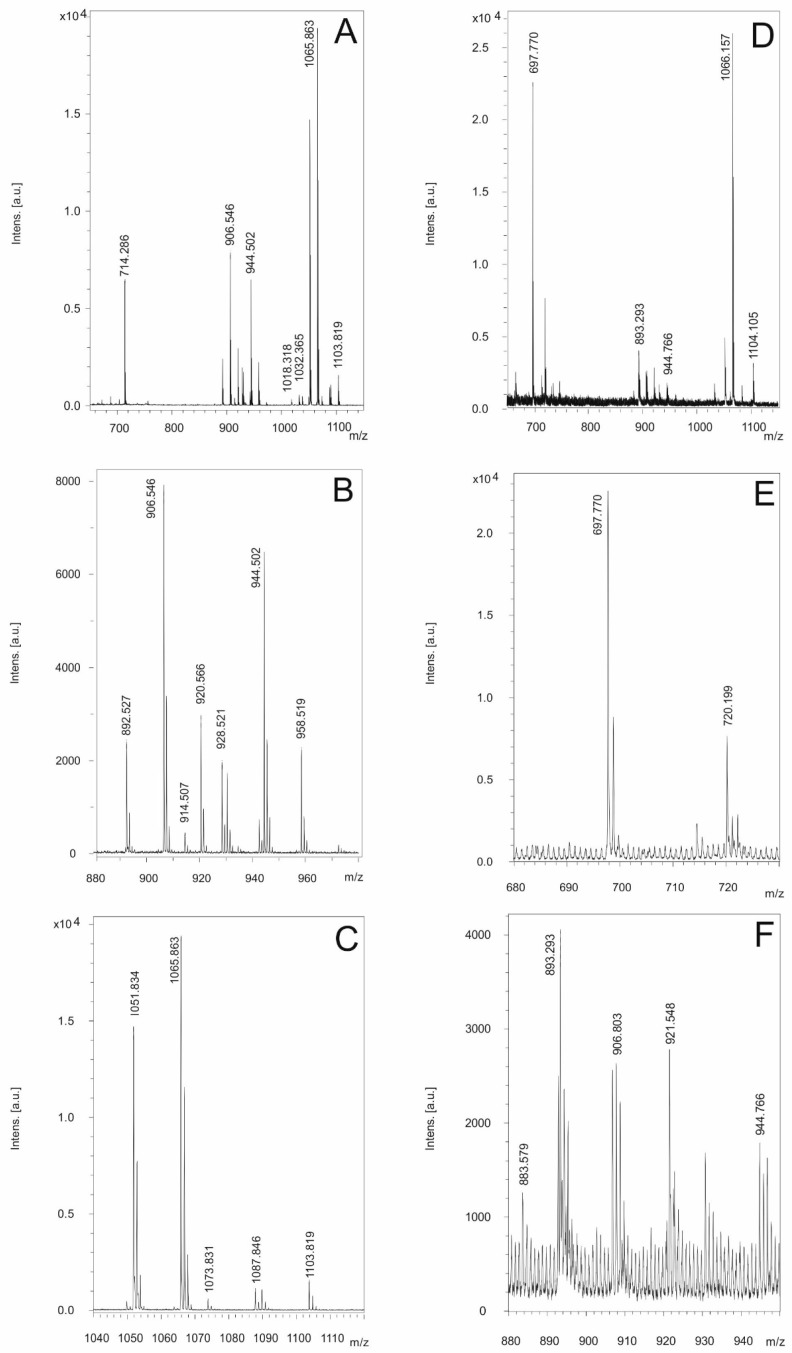
Bioactive compounds produced by *B. cereus* A22. (**A**–**C**) Compounds detected on the surface extracts of this strain. (**A**) MALDI-TOF mass spectrum of a surface extract of strain A22 grown on agar plates in the Landy medium in the mass range of *m/z =* 650–1150. (**B**) Kurstakins observed in the range of *m/z =* 880–980. (**C**) Detection of a yet unknown prominent product with mass numbers of 1051.83 and 1065.86 Da. (**D**–**F**) Compounds found in the culture filtrate of strain A22 grown in the Landy medium for 48 h. (**D**) MALDI-TOF mass spectrum of a culture filtrate of strain A22 grown in the Landy medium for 48 h in the mass range of *m/z =* 690–1120. (**E**) Detection of the arylpolyene-lipopeptide thumolycin and the siderophore petrobactin with mass numbers of 697.77 and 720.20. Da. (**F**) Kurstakins and the siderophore bacillibactin ([M + H;K] ^+^ = 883.58 and 921.55 Da) found in the mass range of *m/z* = 880–950.

The genome mining data summarized in Figure 2 indicate the presence of BGCs at the genomic level. However, the real biosynthetic capacity of the investigated isolates can only be verified by the isolation and structural analysis of the compounds actually produced. In Figure 6, we demonstrate the production of the non-ribosomally formed secondary metabolites of strain A22 as a representative for *B. cereus* detected by MALDI-TOF MS. As an overview, Figure 6A–C show mass spectra for the compounds found in a surface extract of A22 taken from cell materials grown on agar plates in the Landy medium for 48 h. Two prominent products were observed. Figure 6B shows the mass peaks for two kurstakins with chain lengths of their fatty acid component of 12 and 13 carbon atoms, respectively. The following mass data were found: C12-kurstakins: [M + H,Na,K]^+^ = 892,5/914,5/930.5 Da; C13-kurstakins: [M + H,Na,K]^+^ = 906.5/928.5/944.5 Da. In addition, as yet unknown compounds with the mass numbers of 1051.8 and 1065.9 were found, which dominate the MALDI-TOF mass spectrum of the surface extract in Figure 6A. Presumably, there are two isomers that differ by a methylene group (Figure 6C). This compound cannot be correlated to any of the BGCs of the antiSMASH profile of strain A22.

Figure 6D–F show the mass spectra of the products of A22 released into the culture broth for growth in liquid cultures for 48 in the Landy medium. Here, the arylpolyene lipopeptide thumolycin and both siderophores bacillibactin and petrobactin were detected. Figure 6E exhibits the mass peaks of thumolycin (*m*/*z* = 697.8) and petrobactin (*m*/*z* = 720.2). A relatively small part of the kurstakins was released into the culture filtrate, while the main part remained attached at the outer surface of A22. In Figure 6F, kurstakin mass peaks (*m/z* = 906.8/930.8 and 944.8) overlap with those of the siderophore bacillibactin [M + H,Na,K]+ = 883.6/905.6/921 Da. These results demonstrate that kurstakins were predominantly found attached to the outer surface of *B. cereus* cells, while thumolycin and both siderophores bacillibacin and petrobactin were released into the culture medium. Similar profiles were obtained for strains A24, HD1.4B, and HD2.4. All other investigated *B, cereus* isolates did not produce thumolycin.

The structure of both lipopeptide products of strain A22, kurstakin and thumolycin, were investigated in detail by LIFT-MALDI-TOF/TOF fragment analysis [26]. Table 2 shows the sequence determination of C13 kurstakin with a parent ion [M + H}+ = 906.504 Da derived from product ions obtained by LIFT-MALDI-TOF/TOF fragment ion spectra. In Table 3, the structure of this compound is modelled from the nearest neighbor relationships using di-, tri-, and tetrapeptide fragments.

**Table 2:** Mass spectrometric sequence determination of C13-kurstakin produced by *B. cereus* A22 with a parent ion [M + H]^+^ = 906.504 derived from product ion patterns obtained by LIFT-MALDI-TOF/TOF fragment ion spectra. FA: fatty acid component.

b_n_₋H_2_O (*found*)	-	280.123	337.149	408.107	495.159	632.284	760.445	-
b_n_ (*found*)	197.030	298.133	355.112	-	-	-	778.446	906.504
b_n_ (*calc.*)	197.190	298.238	355.259	426.296	513.328	650.387	778.446	906.504
	**C13-FA**	**Thr** (1)	**Gly** (2)	**Ala** (3)	**Ser** (4)	**His** (5)	**Gln** (6)	**Gln** (7)
y_n_ (*calc.*)	906.502	710.322	609.275	552.253	481.216	394.184	257.125	129.066
y_n_ (*found*)	906.504	710.266	609.163	552.133	481.106	394.084	257.050	129.066
y_n_₋H_2_O (*found*)	-	692.185	-	-	463.103	-	-	-

**Table 3:** Modeling of the structure of C13-kurstakin (*m/z* = 906.5) by nearest-neighbor relationships obtained by MALDI-TOF MS.

(A) Dipeptide fragments	*m/z*	*m/z*
	Calc.	found
C13-FA-Thr	298.238	298.141/280.129
Thr-Gly	159.077	159.007/141.022
Gly-Ala	129.066	129.016
Ala-Ser	159.077	159.007/141.022
Ser-His	225.099	225.034/207.019
His-Gln	266.125	266.060
Gln-Gln	257.125	257.050
(B) Tripeptide fragments		
C13-FA-Thr-Gly	341.244	341.170/323.167
Thr-Gly-Ala	230.114	230.026/212.025
Gly-Ala-Ser	216.098	216.024/198.022
Ala-Ser-His	296.136	-/278.050
Ser-His-Gln	353.157	353.084/335.070
His-Gln-Gln	394.184	394.101/335.070
(C) Tetrapeptide fragments		
C13-FA-Thr-Gly-Ala	426.296	-/408.121
Thr-Gly-Ala-Ser	317.146	317.057/299.070
Gly-Ala-Ser-His	353.157	353.084/335.070
Ala-Ser-His-Gln	424.194	-
Ser-His-Gln-Gln	481.216	481.120/463.120

Using the same technique, we investigated thumolycin, which is a combination of a pentapeptide attached to a yet unknown arylpolyene lipidic residue. By LIFT-MALDI-TOF/TOF fragment analysis, we obtained the complete sequence of the pentapeptide part for the first time, which is compatible with the initial results from Zheng et al. [42] and the module organization of the corresponding BGC derived from antiSMASH 6.0 genome mining. The structure of this pentapeptide is shown in Table 4.

In summary, by MALDI-TOF mass spectrometry, we detected all compounds produced by *B. cereus* strains non-ribosomally. The investigation of the RiPPs, such as lanthipeptides, sactipeptides, and bacteriocins, is in still progress.

### 3.4. B. cereus s.l. Strains Suppressed Plant Pathogens and Promoted Plant Growth

#### 3.4.1. Antifungal and Nematocidal Activity

**Figure 7:**
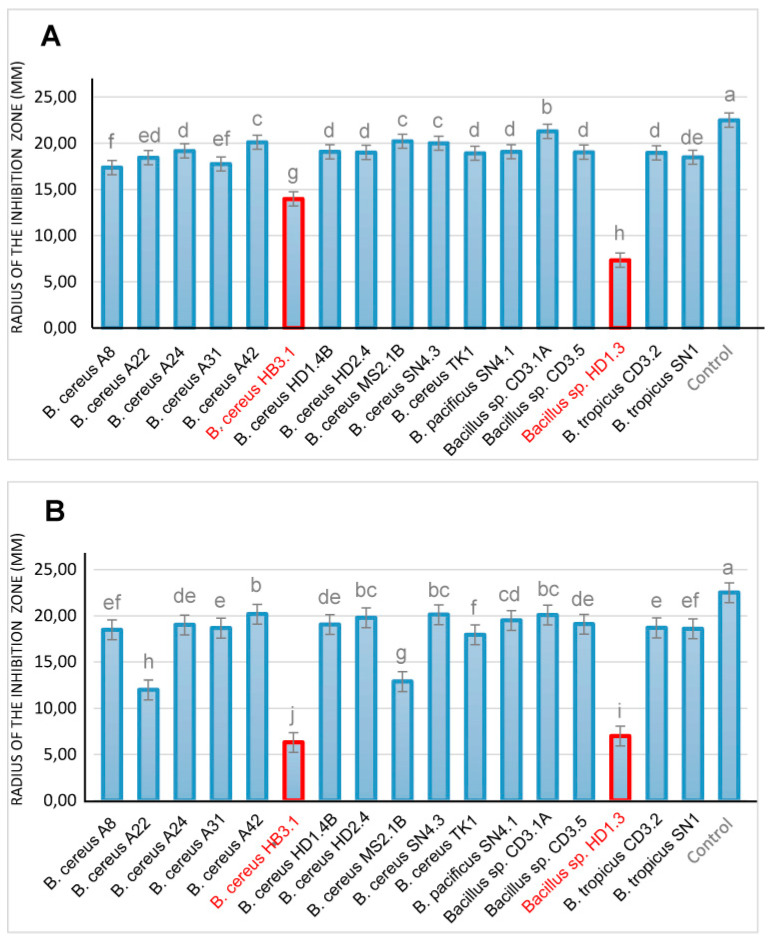
In vitro assay of the antifungal activity of *B. cereus* group strains isolated from Vietnamese crop plants. (**A**) Suppression of *Phytophthora palmivora*. (**B**) Suppression of *Fusarium oxysporum*. Strains with enhanced antimicrobial action are indicated in red. All diagrams show the means of at least three replicates (n ≥ 3). Negative controls were performed without treatment with the bacteria. Columns with superscripts with the same letter are not significantly different according to the Fisher’s least significance difference (LSD) test (*p* ≤ 0.05).

Our in vitro bioassays revealed that only few of the *B. cereus* group isolates efficiently inhibited phytopathogenic fungi and nematodes. Antifungal activity was examined in vitro using *Fusarium oxysporum,* known for causing fusarium wilt disease [80], and *Phytophthora palmivora,* one of the most detrimental plant pathogens in Vietnam [81]. Whilst most of the isolates did not significantly suppress the growth of the pathogenic fungi and oomycetes, a strong antimicrobial activity was exerted by *B. cereus* HB3.1 and *Bacillus* sp. HD.3 (Figure 7).

**Figure 8:**
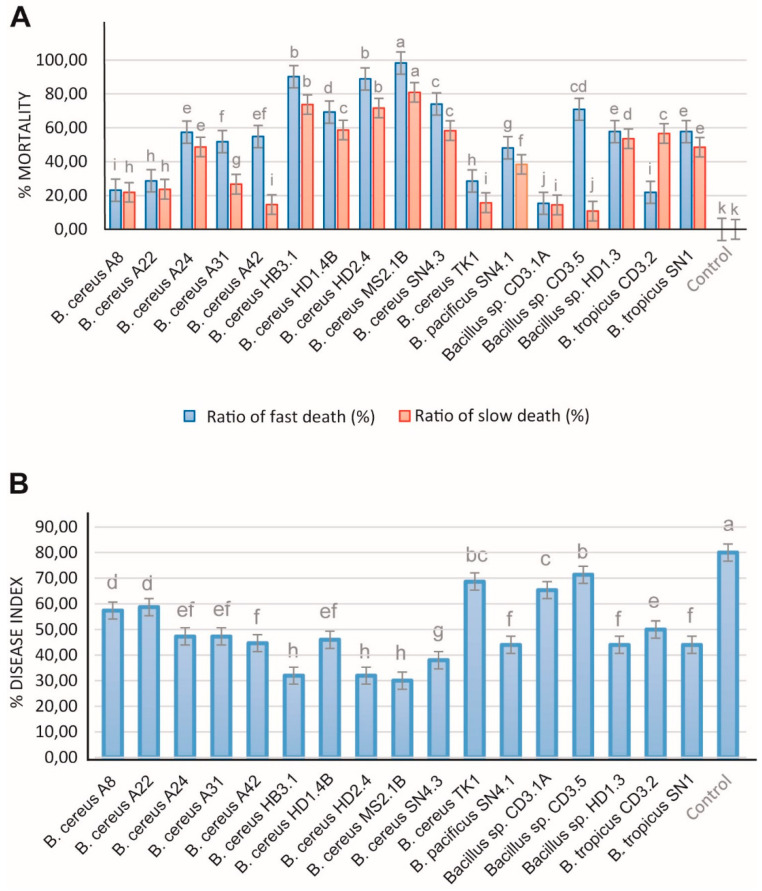
Nematocidal activity of *B. cereus* isolates. (**A**) Bioassay with *Caenorhabditis elegans.* Slow killing activity was determined in NGM plates and fast killing activity in a liquid medium as described previously [3]. (**B**) Determination of the biocontrol action of the *B. cereus* isolates on the root-knot nematode *Meloidogyne* sp. in greenhouse experiments. Tomato plants infested with *Meloidogyne* sp. were used for the test (counting of “knots” in the tomato roots). The increase compared to the control without adding with the *Bacillus* isolates is shown. All experiments were conducted with three independent repetitions and a randomized design. The bars above the columns indicate the standard error (SE). Different letters at each treatment indicate significance between inoculated and uninoculated conditions, at a *p* ≤ 0.05 level after the *t*-test.

Root-knot nematodes, such as *Meloidogyne spp*., are one of the most important plant pathogens in tropical and temperate agriculture, and are responsible for the significant harvest losses of main Vietnamese crops, such as coffee trees (*Coffea arabica* and *Coffea canephora*) and black pepper plants [82]. In order to analyze the antagonistic activity of the *B. cereus* isolates, we tested at first their suppressing effect against the model nematode *Caenorhabditis elegans.* Fast and slow death rates were estimated in a bioassay under laboratory conditions. Most of the investigated *B. cereus* isolates were able to kill considerable amounts of the nematodes, as revealed in both test systems (Figure 8).

In order to examine the inhibiting effects against phytopathogenic nematodes more directly, we isolated a representative strain of *Meloidogyne sp*. from the galls of infested black pepper plant roots according to the hypochlorite procedure [83]. The inhibiting effect exerted by the test bacteria on disease development was examined in a greenhouse experiment. Ten weeks after the transplanting of the tomato plantlets into the soil, the formation of root knots was visually registered and used as a measure for calculating the disease index according to Bridge and Page [28]. All *B. cereus* isolates were found to be efficient against nematodes. In the presence of HB3.1, HD1.4B, HD2.4, MS2.1B, and SN4.3, the killing rates (estimated as slow and fast killing rates) of *Caenorhabditis elegans* exceeded 60% (Figure 8A). The *Melodoine* sp.-caused disease index of tomato plants was reduced by more than 50% after the application of *B. cereus* HB3.1, HD2.4, MS2.1B, and SN4.3 (Figure 8B). Similar rates were previously detected in representatives of *B. velezensis* [4] and *Brevibacillus* spp. [3].

#### 3.4.2. Plant Growth Promotion

**Figure 9:**
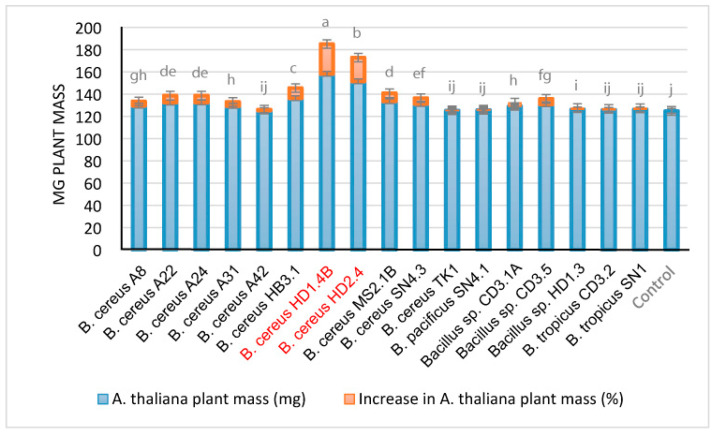
Growth-promoting effects of *B. cereus* isolates on *Arabidopsis thaliana* seedlings. The blue columns in the diagram represent the fresh weight obtained after 21 days under controlled conditions in the growth chamber. The % increase compared to the untreated control (red columns) is indicated on top of the columns. Each treatment value is presented as the means of three replications (n = 3) with the standard error. Different letters at each treatment indicate significance between inoculated and uninoculated conditions, at a *p* ≤ 0.05 level after the *t*-test.

We examined the effect of the Vietnamese *B. cereus* isolates on the *Arabidopsis thaliana* biotest system [29]. *B. cereus* HD1.4B and *B. cereus* HD2.4 enhanced the growth of the *Arabidopsis* seedlings by more than 20% (Figure 9). However, using the same biotest system, the increase rates observed for some plant-associated *Brevibacillus* and *B. velezensis* strains isolated during the same survey [2] were higher and estimated to range from 30 to 40% [3,4].

## 4. Conclusions

In this study, we showed that plant-associated representatives of the *B. cereus* group were able to suppress important plant pathogens, such as fungi (*Fusarium oxysporum*), oomycetes (*Phytophthora palmivora*), and root-knot forming nematodes (*Meloidogyne* sp.) The plant-growth-promoting activity of some of the isolates could also be demonstrated.

Genome mining revealed that the members of the *B. cereus* group are rich in gene clusters probably involved in the synthesis of antimicrobial peptides that are efficient in inhibiting plant pathogens and triggering plant-induced systemic resistance [84]. A total of 36 different biosynthetic gene clusters (BGCs), many of them not listed in the MiBIG data bank, were detected in the 17 isolates obtained from Vietnames crop plants. Mass-spectrometric analysis revealed that, in addition to some hitherto unknown compounds, several species of the antimicrobial lipopeptides kurstakin and thumolycin and the siderophores bacillibactin and petrobactin were expressed in many of the isolates. The arylpolyene lipopeptide thumolycin was reported to possess interesting antimicrobial and nematocidal activities, but its primary structure was not resolved [42]. Here, the primary structure of the pentapeptide part was resolved, and the Orn residue was identified as being linked with the yet unknown arylpolyene lipid part.

In addition to antimicrobial peptides, the biocontrol of plant pathogens can be exerted by δ-endotoxins (parasporal inclusion proteins), traditionally known to be produced by *B. thuringiensis*, a close relative of *B. cereus*. *Cry* genes encoding the entomocidal crystal proteins Cry1Aa1 and Cry2Ba1 were detected in the genome of *B. cereus* ssp. *Bombysepticus* TK1. In the same strain, another gene encoding the vegetative insecticidal protein Vip3 was also detected, suggesting that the presence of insecticidal proteins is not restricted to *B. thuringiensis*. The encouraging results described above might lead to the development of the selected representatives of the *B. cereus* group as biocontrol agents. However, as known food poisoning organisms and members of the risk group 2, the potential of the *B. cereus* group isolates to produce toxins needs to be carefully examined before they can be applied in sustainable agriculture.

In this context, we showed that no gene clusters encoding *B. anthracis* pXO plasmid-related toxins were present in all the *B. cereus s.l*. genomes investigated here. Furthermore, we could rule out the synthesis of the heat-stable cereulide toxin, the causative agent of the emetic syndrome. However, the regular appearance of chromosomally localized virulence genes, encoding the heat-labile enterotoxins HBL and NHE, might restrict the direct application of *B. cereus s.l*. strains in biological plant protection.

In order to avoid such conflicts, the utilization of interesting *B. cereus* BGCs and genes encoding entomocidal proteins can be achieved by their heterologous expression in safe plant-beneficial host strains, which has been already demonstrated with *B. velezensis* FZB42 [85,86].

## Figures and Tables

**Table 4:** Sequence of the pentapeptide part of the lipopeptide thumolycin derived from the product ion pattern obtained by LIFT-MALDI-TOF/TOF fragment ion spectra. The yet unknown arylpolyene lipid part of thumolycin of unknown length is linked to the Orn residue. The amino acids Orn, Ile, and Leu were also predicted by their adenylation domain sequences from genome mining using antiSMASH 6.0 (Figure 3B).

b ions →					
b_n_ *(found)*	**-**	216.17	328.25	457.38	(p)??
b_n_ *(calc.)*	115.09	216.14	329.22	457.28	(P)570.36
	**Orn** (1)	**Thr** (2)	**Ile** (3)	**Gln** (4)	**Leu** (5)
y_n_ *(calc.)*	(p)570.36	456.28	355.24	242.15	114.09
y_n_ *(found)*	**(p)??**	**456.39**	**355.25**	**242.17**	**-**
					**<--** y ions

## Data Availability

Gene bank accession numbers of the complete genome sequences available in the NCBI data bank are listed in Section 2, Materials and Methods.

## Supplementary Materials

The following supporting information can be downloaded at: https://www.mdpi.com/article/10.3390/microorganisms11112677/s1. Figure S1: Tree inferred with FastMe 2.1.6.1 from GBDP distances calculated from 16S rDNA gene sequences. Figure S2: GBDP tree (whole-genome-sequence-based) inferred with FastMe 2.1.6.1 from GBDP distances calculated from genome sequences. Figure S3: Heat map of the FastANI values of 128 *B. cereus* group genomes calculated and drawn by the EDGAR3.0 software package. Figure S4: Environment of the Rep protein genes in the plasmid sequences of A22, A24, HD1.4B, and HD2.4; Figure S5: Localization of the virulence genes and gene clusters on the chromosomes and plasmids of *B. cereus* isolates. The *cytK* gene and the NHE/HBL gene clusters were chromosomally localized. The complete set of NHE and HBL genes was chromosomally localized in all four completely sequenced strains (A22, A24, HD1.4B, and HD2.4). The P1 plasmid sequences of HD1.4B and HD2.4 harbored genes with similarity to the NHE/HBL enterotoxin family. Figure S6: Plasmid-encoded catabolic operons, biosynthetic gene clusters (BGCs), and restriction/modification systems. Figure S7: BGCs in the *B. cereus* group isolates encoding NRPS/PKS and other secondary metabolites. Figure S8: RiPP gene clusters detected by applying the BAGEL4 software (http://bagel4.molgenrug.nl/ (accessed on 1 November 2021)) in the genomes of the Vietnamese *Bacillus cereus* group genomes. Figure S9: RiPP gene clusters detected by applying the antiSMASH version 6 software in the Vietnamese *Bacillus cereus* group genomes. Table S1: Types of entomocidal cry toxins. Table S2: Species and subspecies delineation of the 17 Vietnamese *B. cereus* sensu lato isolates according to their dDDH and Fast ANI values. Table S3: List of the 17 *B. cereus* group strains used in this study with detailed annotation. Table S4: Genome annotation of the four completely sequenced B. cereus strains by RASTk. Table S5: Gene clusters (BGCs) of the Vietnamese Bacillus cereus group isolates. Table S6: Gene clusters (BGCs) of the Vietnamese Bacillus cereus group isolates involved in the non-ribosomal synthesis of antimicrobial peptides (RiPPs and bacteriocins). Table S7: Gene clusters (BGCs) of the Vietnamese Bacillus cereus group isolates involved in the ribosomal synthesis of antimicrobial peptides (RiPPs and bacteriocins). Table S8: Gene clusters (BGCs) of the Vietnamese Bacillus cereus group isolates involved in the synthesis of other secondary metabolites.
