# Supplementary material for: Plant-Associated Representatives of the Bacillus cereus Group Are a Rich Source of Antimicrobial Compounds
Source: Microorganisms. 2023 Oct 31;11(11):2677. doi: 10.3390/microorganisms11112677 (PMC10672896; doi:10.3390/microorganisms11112677)
Supplement: Supplementary file 1 [file microorganisms-11-02677-s001.zip › Suppl. Fig. 1-2, 4-9.pdf]

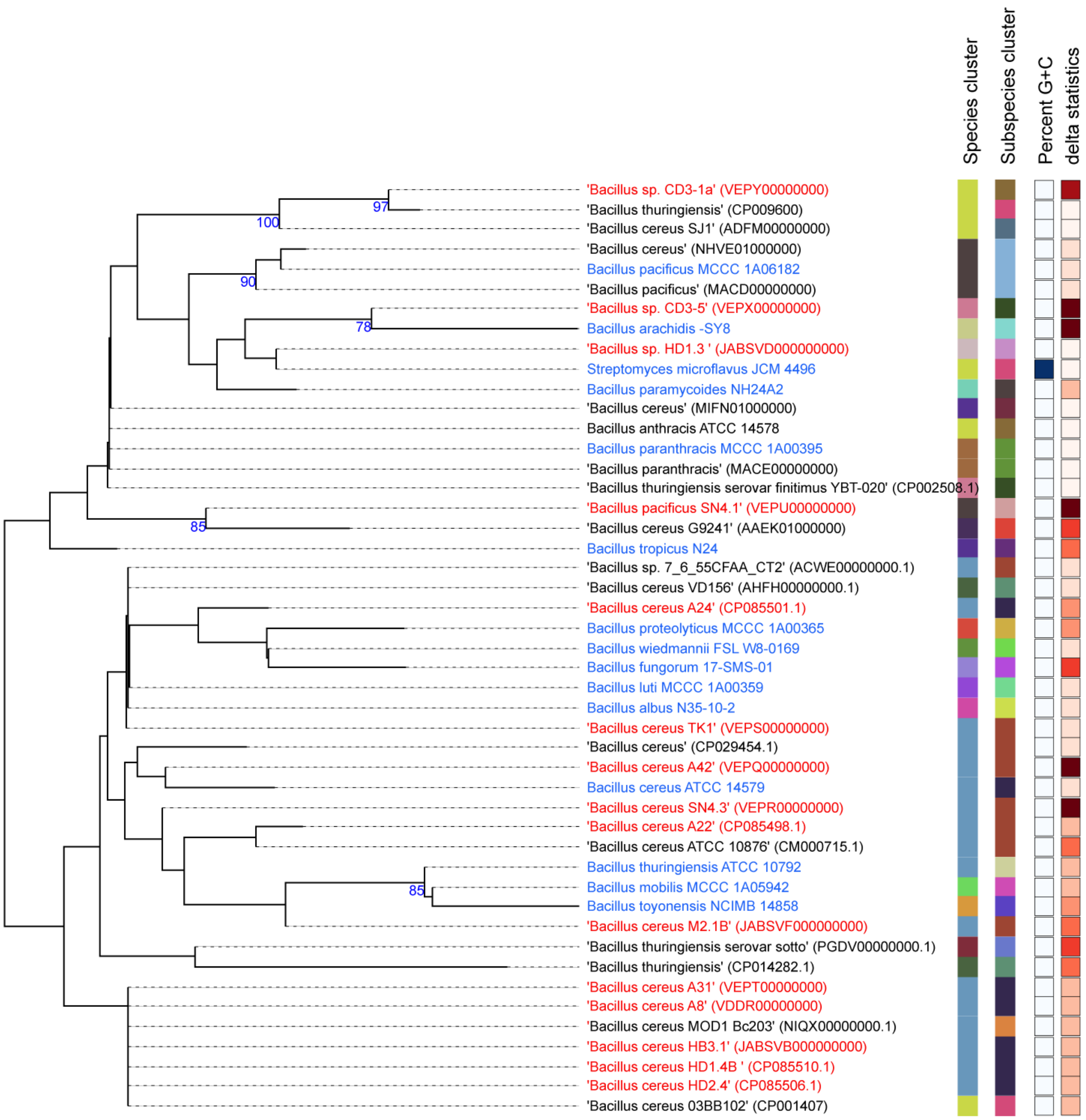

Suppl. Figure S1: Tree inferred with FastMe 2.1.6.1 from GBDP distances calculated from 16S rDNA gene sequences. The branch lengths are scaled in terms of GBDP distance formula d_5. The numbers above branches are GBDP pseudo-bootstrap support values >60% from 100 replications, with an average branch support of 28.5%. The tree was rooted at the midpoint. The 17 Vietnamese crop plant isolates are indicated by red letters. Type strains are indicated by blue letters.

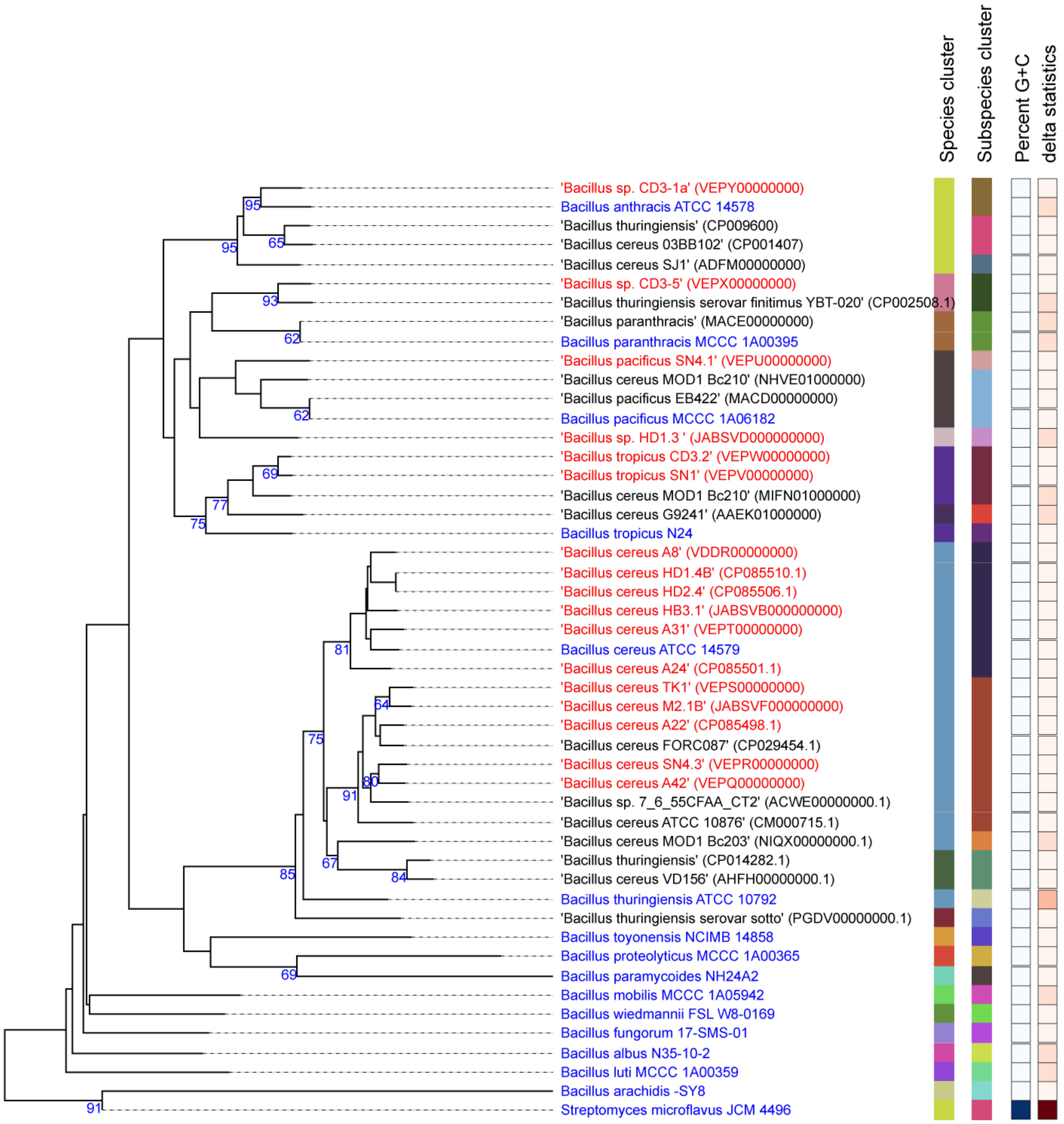

Suppl. Figure S2. GBDP tree (whole genome sequence based) inferred with FastMe 2.1.6.1 from GBDP distances calculated from genome sequences. The branch lengths are scaled in terms of GBDP distance formula d_5. The number above branches are GBDP pseudo-bootstrap support values > 60% from 100 replications, with an average branch support of 52.7%. The tree was rooed at the midpoint. The 17 Vietnamese crop plant isolates are indicated by red letters. Type strains are indicated by blue letters.

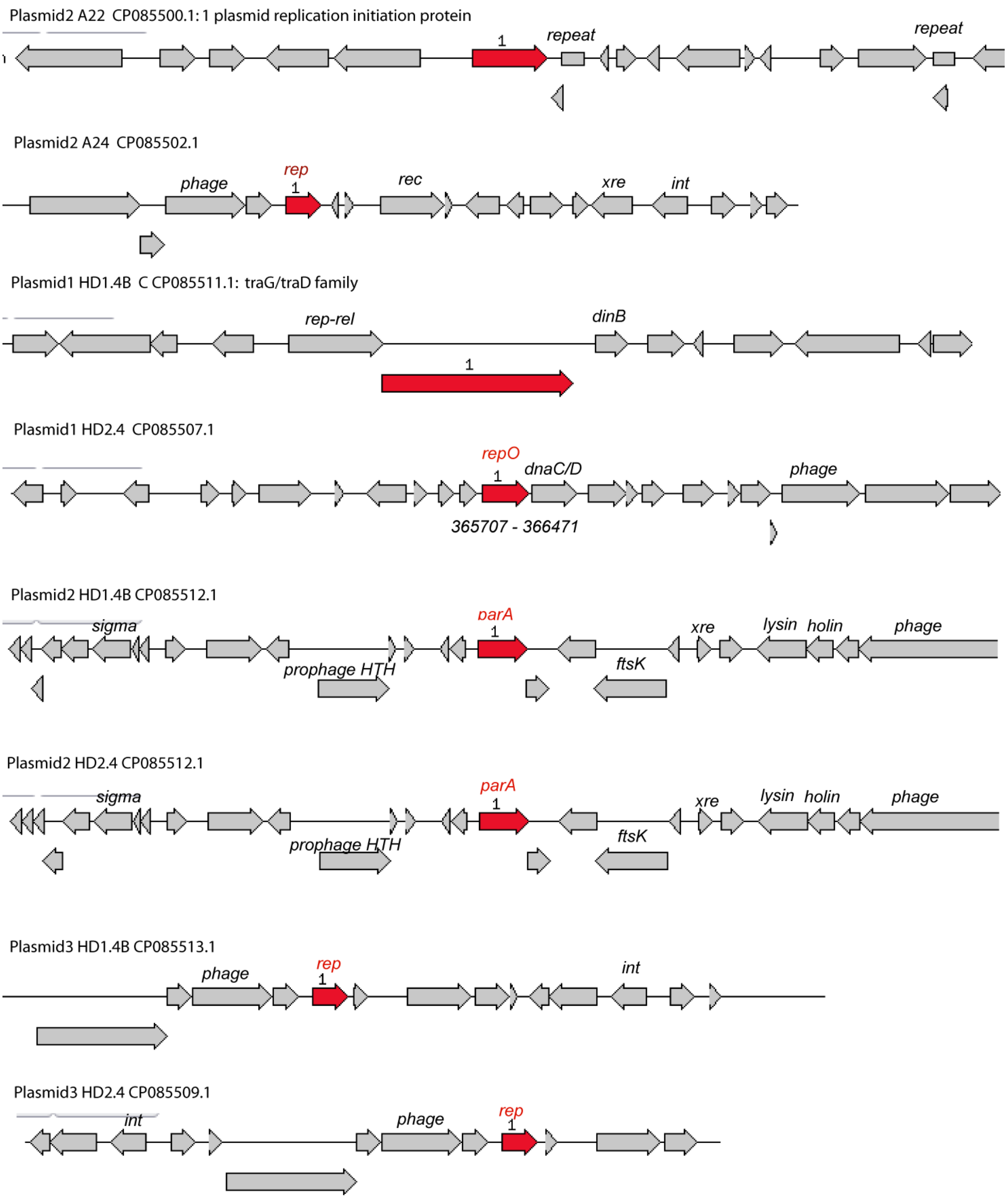

Suppl. Figure S4. Environment of the Rep protein genes in the plasmid sequences of A22, A24, HD1.4B, and HD2.4.

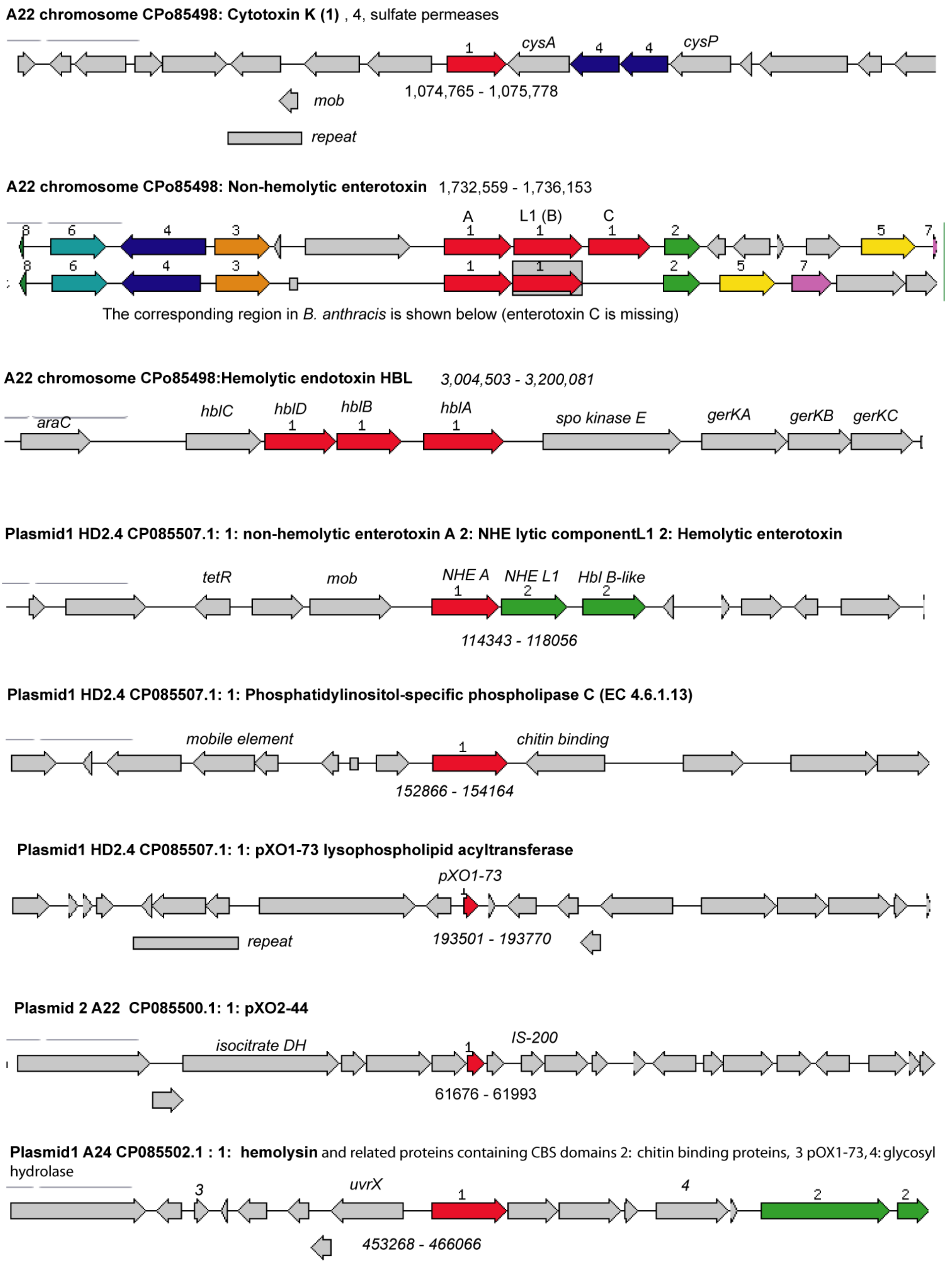

Suppl. Figure S5. Localization of virulence genes and gene clusters on chromosomes and plasmids of B. cereus isolates. The cytK gene and the NHE/HBL gene clusters were chromosomally localized. The complete set of NHE and HBL genes was chromosomally localized in all four completely sequenced strains (A22, A24, HD1.4B, HD2.4). The P1 plasmid sequences of HD1.4B and HD2.4 harbored genes with similarity to the NHE/HBL enterotoxin family.

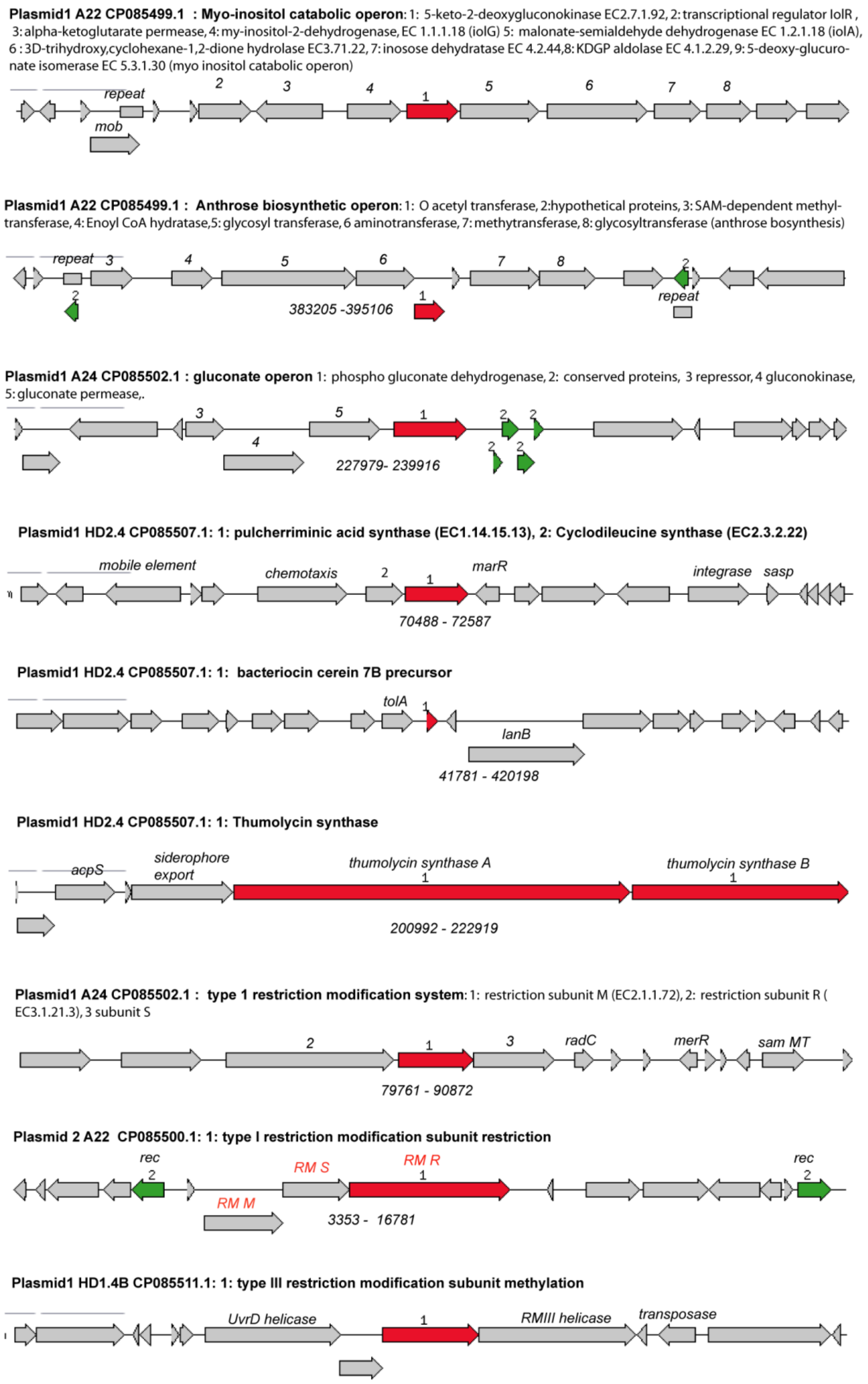

Suppl. Figure S6: Plasmid encoded catabolic operons, biosynthetic gene clusters (BGCs) and restriction/modification systems.

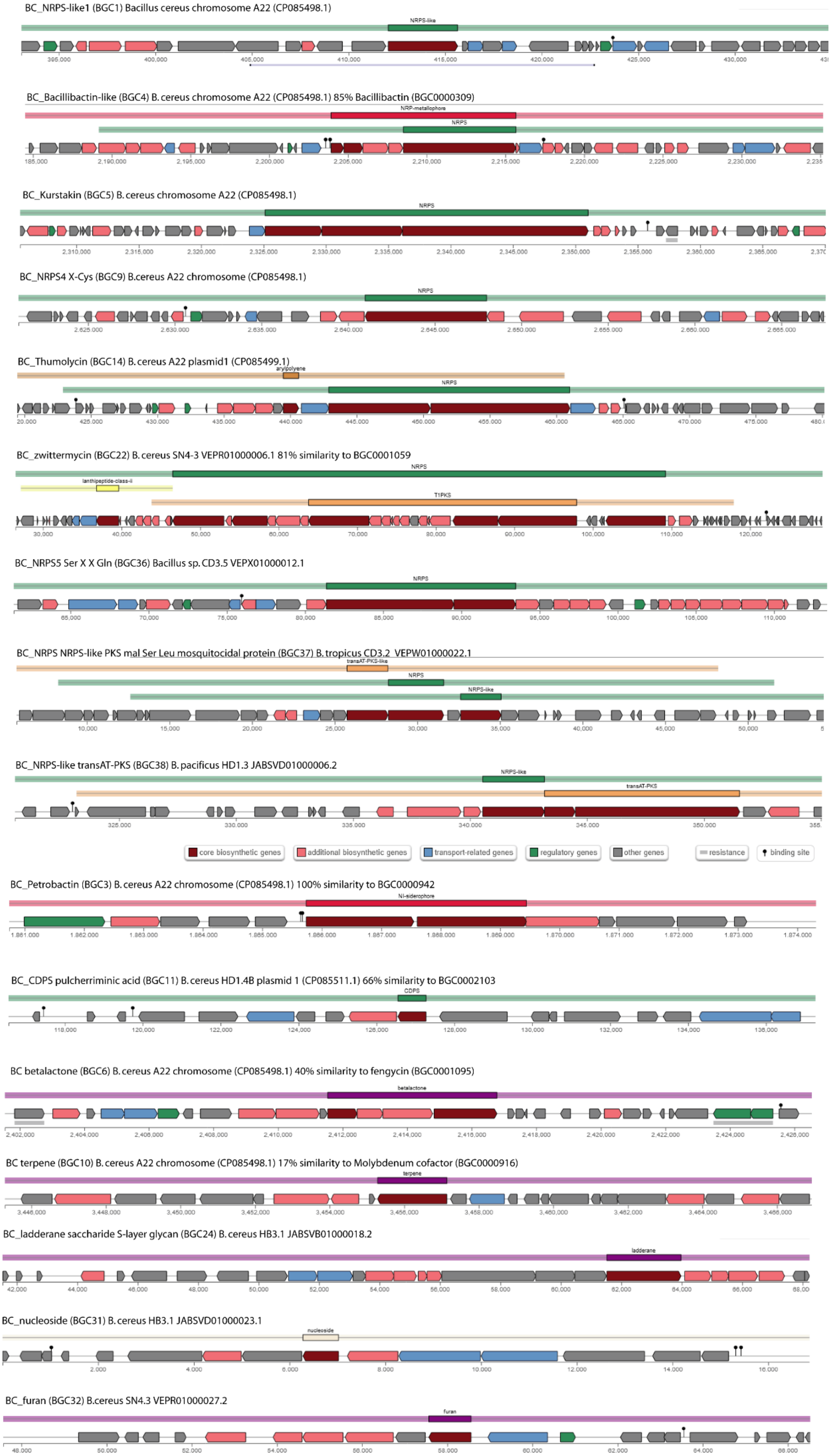
BC_NRPS-like1 (BGC1) Bacillus cereus chromosome A22 (CP085498.1)
NRPS-like
BC_Bacillibactin-like (BGC4) B. cereus chromosome A22 (CP085498.1) 85% Bacillibactin (BGC0000309)
NRP-metallophore
NRPS
BC_Kurstakin (BGC5) B. cereus chromosome A22 (CP085498.1)
NRPS
BC_NRPS4 X-Cys (BGC9) B.cereus A22 chromosome (CP085498.1)
NRPS
BC_Thumolycin (BGC14) B. cereus A22 plasmid1 (CP085499.1)
arylpolyene
NRPS
BC_zwittermycin (BGC22) B. cereus SN4-3 VEPR01000006.1 81% similarity to BGC0001059
NRPS
lanthipeptide-class-ii
T1PKS
BC_NRPS5 Ser X X Gln (BGC36) Bacillus sp. CD3.5 VEPX01000012.1
NRPS
BC_NRPS NRPS-like PKS mal Ser Leu mosquitocidal protein (BGC37) B. tropicus CD3.2 VEPW01000022.1
transAT-PKS-like
NRPS
NRPS-like
BC_NRPS-like transAT-PKS (BGC38) B. pacificus HD1.3 JABSVD01000006.2
NRPS-like
transAT-PKS
core biosynthetic genes
additional biosynthetic genes
transport-related genes
regulatory genes
other genes
resistance
binding site
BC_Petrobactin (BGC3) B. cereus A22 chromosome (CP085498.1) 100% similarity to BGC0000942
NI-siderophore
BC_CDPS pulcherriminic acid (BGC11) B. cereus HD1.4B plasmid 1 (CP085511.1) 66% similarity to BGC0002103
CDPS
BC betalactone (BGC6) B. cereus A22 chromosome (CP085498.1) 40% similarity to fengycin (BGC0001095)
betalactone
BC terpene (BGC10) B. cereus A22 chromosome (CP085498.1) 17% similarity to Molybdenum cofactor (BGC0000916)
terpene
BC_ladderane saccharide S-layer glycan (BGC24) B. cereus HB3.1 JABSVB01000018.2
ladderane
BC_nucleoside (BGC31) B. cereus HB3.1 JABSVD01000023.1
nucleoside
BC_furan (BGC32) B.cereus SN4.3 VEPR01000027.2
furan

Suppl. Figure S7. BGCs in the *B. cereus* group isolates encoding NRPS/NRPK and other secondary metabolites

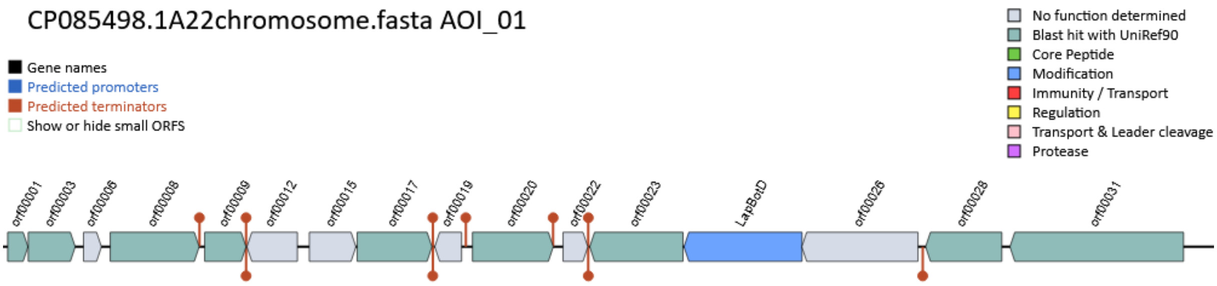

The LAPBotD gene cluster located within the A8 node 12, the A22 chromosome from 1,203,959 – 1,223,959, the A24 chromosome from 4,003,443-4,009,306.

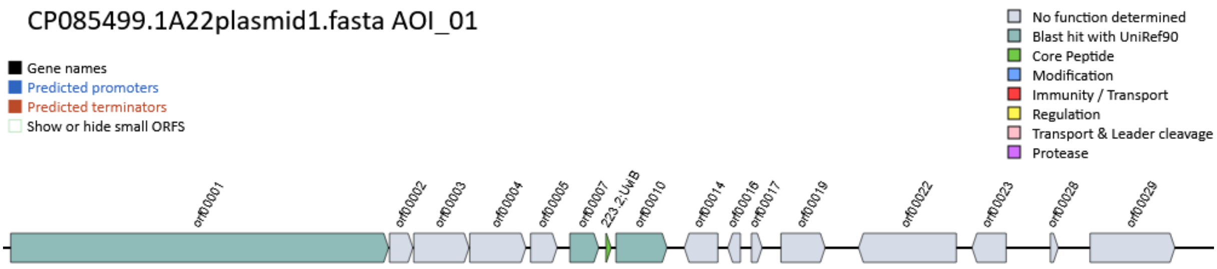

UviB (223.2) located within the A22 chromosome from 350679– 350766. The UviB core peptide sequence is: MLFITQKKNEQCEEQYQAVIQKNQEVIEE

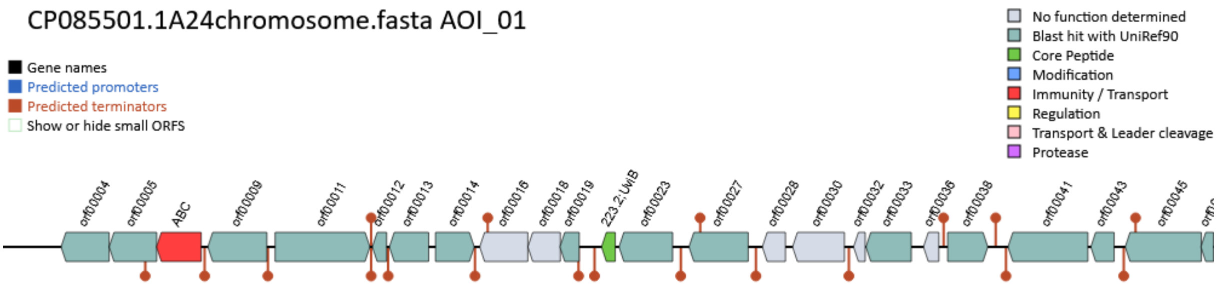

UviB (223.2) located within the A24 chromosome from 1,060,832-1081057. The UviB prepeptide sequence MEEQIFNSMIQQGAFAALFVWMLFTTQKKNEQREEQYQKVIEKNQQVIEEQAKAFSSLSKDLSDVKRKILGNDDEK is identical with Bacillus thuringiensis serovar israelensis ATCC35

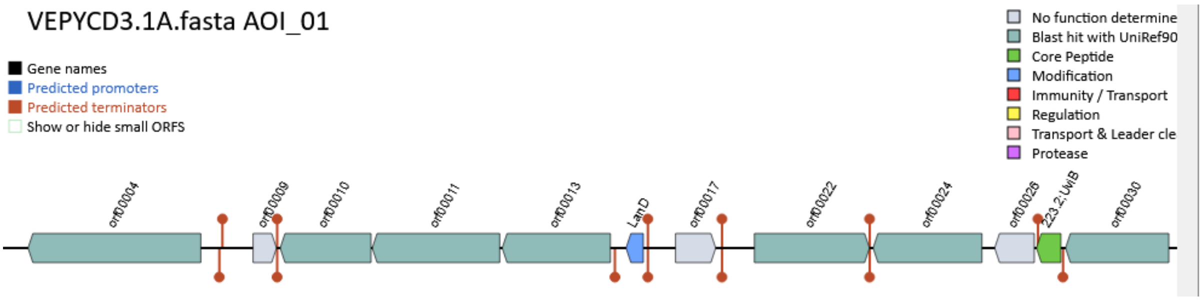

UviB (223.2)/LanD located in Bacillus sp. CD3.1A VEPY 12.1.

```
Query VKTVEEQIFNSMIQQGAFAALFVWMLFTTQKKNEQREAQYQAVIQKNQEVIEEQAKAFGSISKDVTEIKQQIFAD
         +EEQIFNSMIQQGAFAALFVWMLFTTQKKNEQRE QYQ VI+KNQ+VIEEQAKAF S+SKD++++KQ+I  +
UviB     MEEQIFNSMIQQGAFAALFVWMLFTTQKKNEQREEQYQKVIEKNQQVIEEQAKAFSSLSKDLSDVKQKILGNGDEK
```

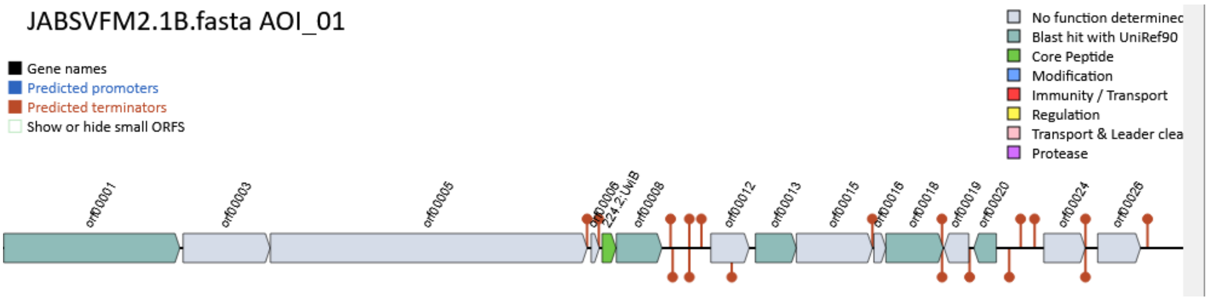

UviB (224.2) located within B. cereus M2.1B JABSVF 20. Weak similarity to *B. thuringiensis sv israelensis* ATCC35.

```
Query MLEQLAQVGLKEGIFALLFIWLLVDTKKESKEREDKLYNFLDGMKDEFSKLVHNYESLSSDVEDIKNDI
      M EQ+    +++G FA LF+W+L  T+K++++RE++    ++  +D  +K    +  LS DV +IK  I
UviB  MEEQIFNSMIQQGAFAALFVWMLFTTQKKNEQREEQYQKVIEKNQDVITKQAEAFGDLSKDVSEIKQKILGSGDVQ
```

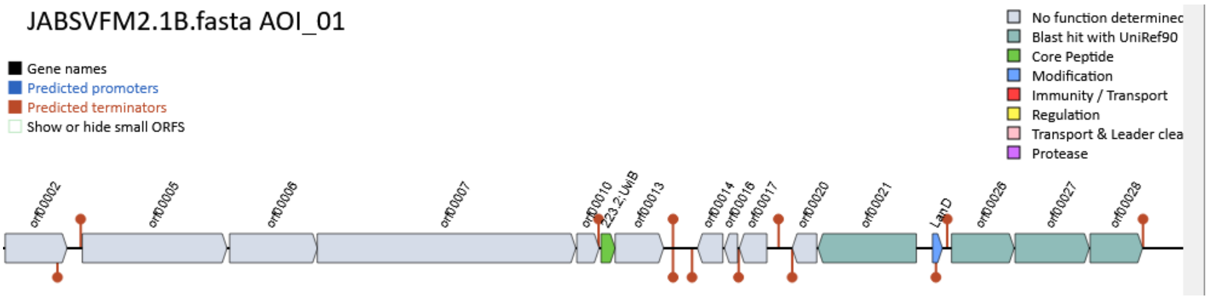

UviB (223.2) located within B. cereus M2.1B JABSVF 1. Similar to B. thuringiensis sv israelensis ATCC35.

```
Query MRTVEDAIFNSVIQQGAFAALFVWMLFTTQKKNEQREEKYQQVIDRNQQVIEEQAKAFGSISKDVTEIKQKLF-EGD
         +E+ IFNS+IQQGAFAALFVWMLFTTQKKNEQREE+YQ+VI++NQQVIEEQAKAF S+SKD++++KQK+   GD
UviB     MEEQIFNSMIQQGAFAALFVWMLFTTQKKNEQREEQYQKVIEKNQQVIEEQAKAFSSLSKDLSDVKQKILGNGDEK
```

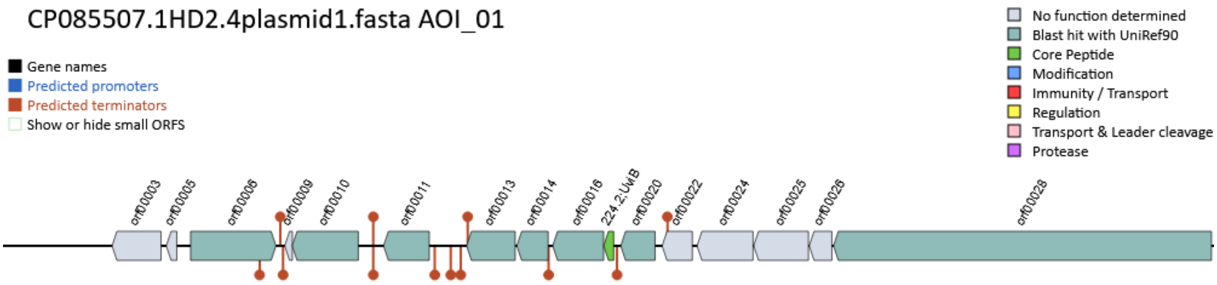

BhlA/UviB (224.2) holin like peptide [WP_098328186.1] in HD2.4 plasmid 1. Weak similarity with Bacillus thuringiensis serovar israelensis ATCC35

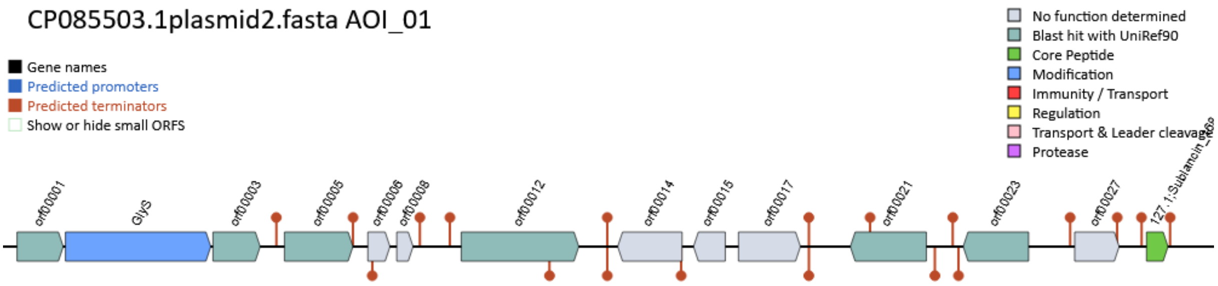


```
Query         MKDLFKELKVEELDKHTGHGGMGWAQCAALLAQCSSGGRIGCGGTATQAYGQCNTYRKMC
              M+ LFKE+K+EEL+    G  G+G AQCAAL   QC+SGG IGCGG A       C   YR+ C
Sublancin_168 MEKLFKEVKLEELENQKG-SGLGKAQCAALWLQCASGGTIGCGGGAV----ACQNYRQFCR
Bridges
Bridges
```

Sublancin 168 (ComC, subclass glyocin) PF03047 in A24-plasmid 2 from 1,652-12,241.
GlyS: SP beta glycosyltransferase SunS.

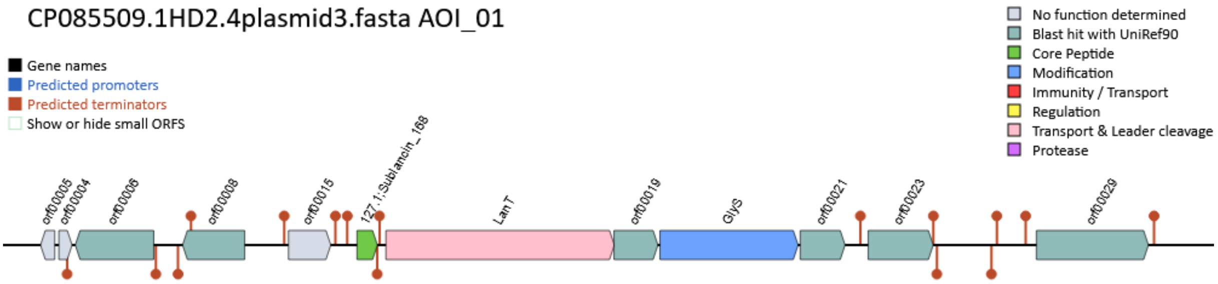

Sublancin 168 (ComC, subclass glyocin) P68577
/LanT in HD1.4B plasmid 3.

```
Query         MKDLFKELKVEELDKHTGHGGMGWAQCAALLAQCSSGGRIGCGGTATQAYGQCNTYRKMC
              M+ LFKE+K+EEL+    G  G+G AQCAAL   QC+SGG IGCGG A       C   YR+ C
Sublancin_168 MEKLFKEVKLEELENQKG-SGLGKAQCAALWLQCASGGTIGCGGGAV----ACQNYRQFCR
Bridges
Bridges
```

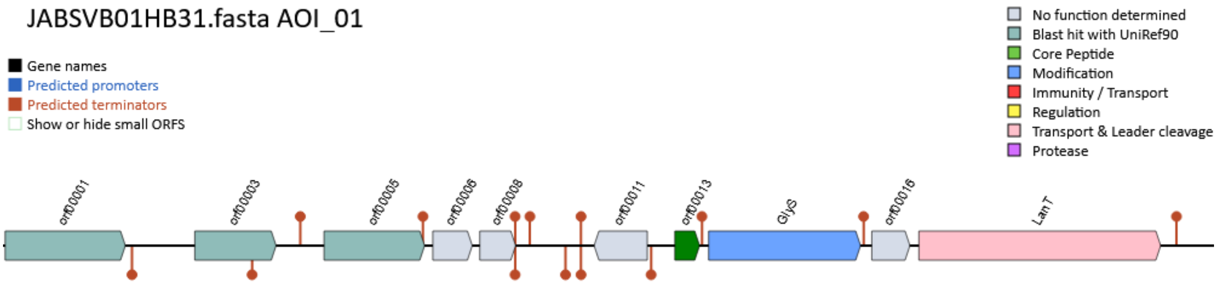

ComC/ GlyS/LanT gene cluster In HB3.1 29.1.

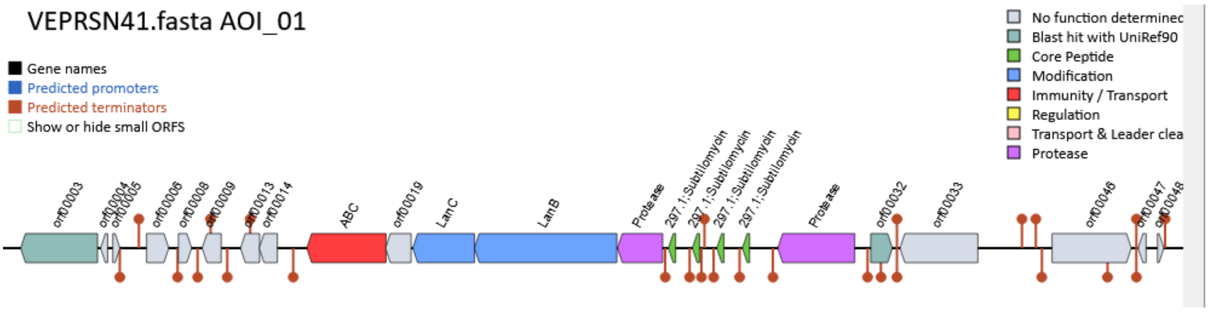

Subtilomycin (297.1)/LanB/LanC gene cluster in SN4-3 VEPR 27.1.

Query MNKELFDLDINKKMETPTEMTAQTWTTIVKV---SKAVCKTGTCICT-TSCSNC

+FDLDINKKME+ +E++AQTW TI K S C+T TC C+ SCSNC

Subtilomycin MEKNNIFDLDINKKMESTSEVSAQTWATIGKTIVQSVKKCRTFTCGCSLGSCSNCN

Subclass Lanthipeptide

Organism Bacillus subtilis

Literature Reference

NCBI JX912247.1

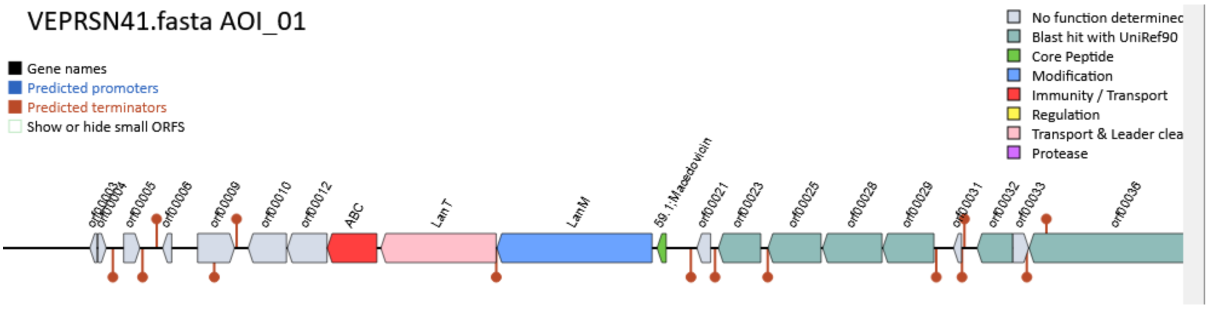

Macedovicin (59.1)/LanM/LanT gene cluster in SN4-3 VEPR 6.2.

Query METEKYLQVVEDEEIEQLVGGVGPGWIETLTKDCPGYKPYACITIAGQTI--CKRC

E + +++ V D+E+E L+GG GWI+TLTKDCP C AG I CK C

Macedovicin MMNATENQIFVETVSDQELEMLIGGADRGWIKTLTKDCPNVISSIC---AGTIITACKNCA

Bridges

Bridges

Bridges

Modifications MMNATENQIFVETVSDQELEMLIGGADRGWIK*L*KDCPNVISSIC---AGTIITACKNCA

Subclass Lanthipeptide B

Organism Streptococcus macedonicus ACA-DC198

Literature Reference

UniProt H2A7G5

propeptide 1 - 25

chain Lantibiotic macedovicin 26 - 58

modified residue 2,3-didehydrobutyrine 33

modified residue 2,3-didehydrobutyrine 35

disulfide bond 46 - 54

cross-link Beta-methyllanthionine (Thr-Cys) 33 - 38

cross-link Beta-methyllanthionine (Thr-Cys) 35 - 57

The macedovicin peptide was found identical to bovicin HJ50 and thermophilin 1277.

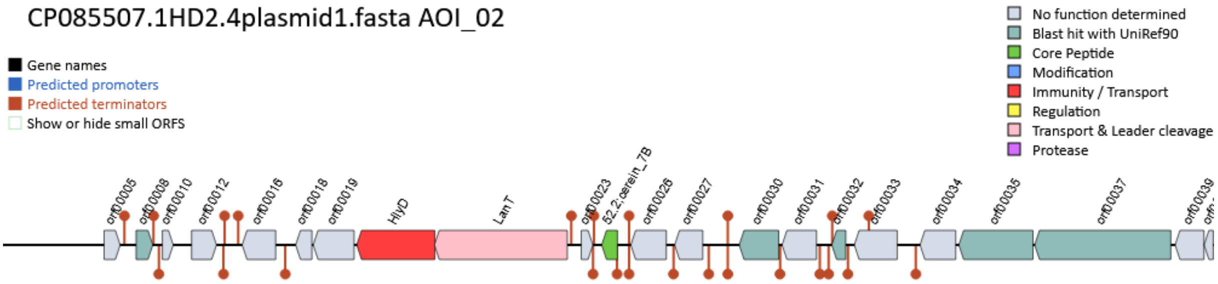

Cerein_7B (52.2) Q2MDB2

LanT gene cluster in HD2.4 plasmid 1 from 409,978-430,197

HlyD putative bacteriocin ABC transporter. Cerein_7B ComC; L_biotic_typeA, bacteriocin_IlC.

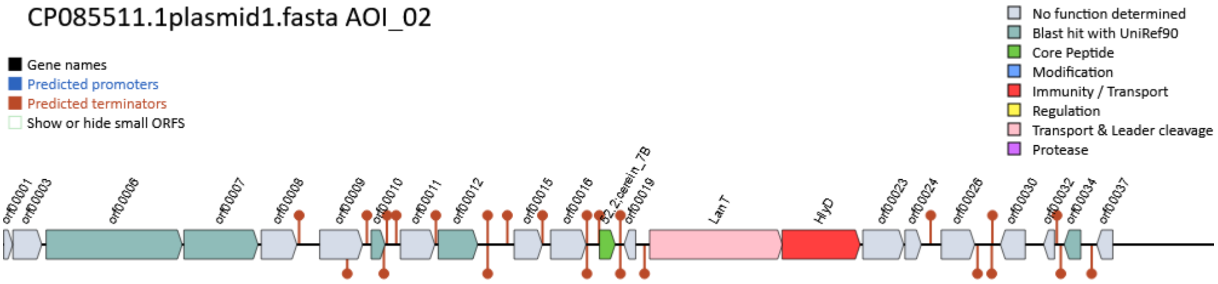

Cerein_7B (subclass D) gene cluster in HD1.4B) plasmid1 from 270,830-291,049. Q2MDB2. Similar to B. cereus

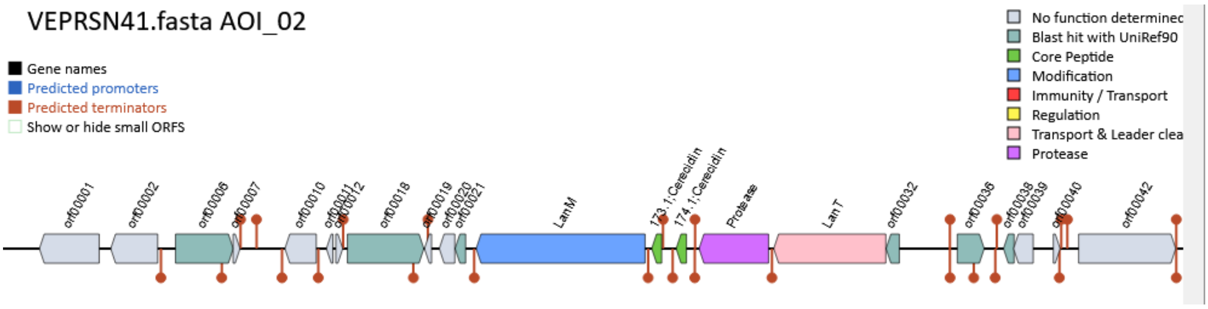

Cerecidin (173.1, 174.1) gene cluster in VEPR 6.1 SN4-3.

Query MNRNQVIEELAVNHPAGAKLVEVSREELTRVYGGGDVQAETTPMTPTLYLNGITIGLALSKQSC

NHP+G L E+S EEL + G DVQ ETTP+ G+ IG+ S + C

Cerecidin MSKGYKFTKEELVEAWKDPQVREKLKDLPNHPSGKALNELSEEELAEIQGASDVQPETTPLC-----VGVIIGITASIKICK

Subclass Lanthipeptide B

Organism Bacillus cereus

Literature Reference

NCBI AHJ59543.1

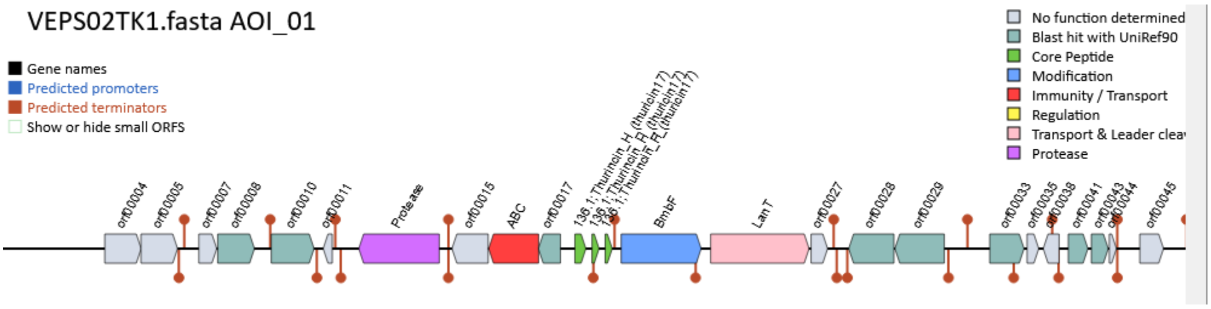

Sactipeptide Thurincin_H (thuricin 17)/BmbF//LanT gene cluster in M2.1B, JABSVF 11.1, TK1 VEPS 63.1

Query METPVVQPRDWTCWSCLVCAACSVELLNLVTAATGASTAS

METPVVQPRDWTCWSCLVCAACSVELLNLVTAATGASTAS

Thurincin_H_(thuricin17) METPVVQPRDWTCWSCLVCAACSVELLNLVTAATGASTAS

Subclass Sactipeptide

Organism Bacillus thuringiensis

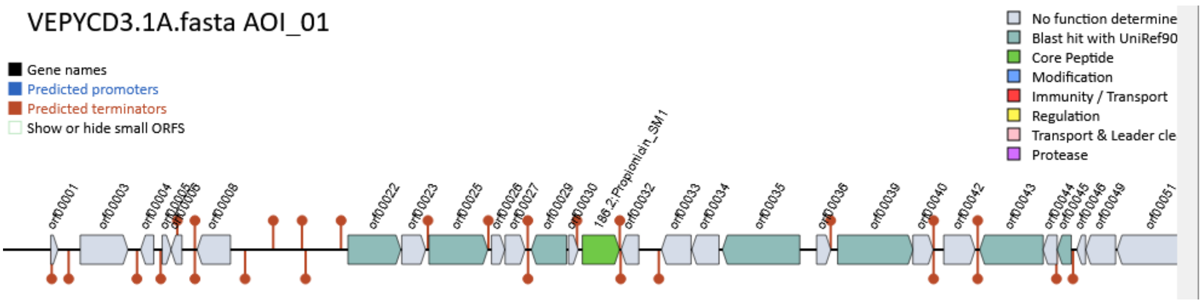

Propionicin (186.2) in Bacillus sp. CD3.1A VEPY 16.1. Weak similarity to Propionicin

Miescher S, Stierli MP, Teuber M, Meile L. Propionicin SM1, a bacteriocin from Propionibacterium jensenii DF1: isolation and characterization of the protein and its gene. Syst Appl Microbiol. 2000 Jun;23(2):174-84. doi: 10.1016/S0723-2020(00)80002-8. PMID: 10930068.

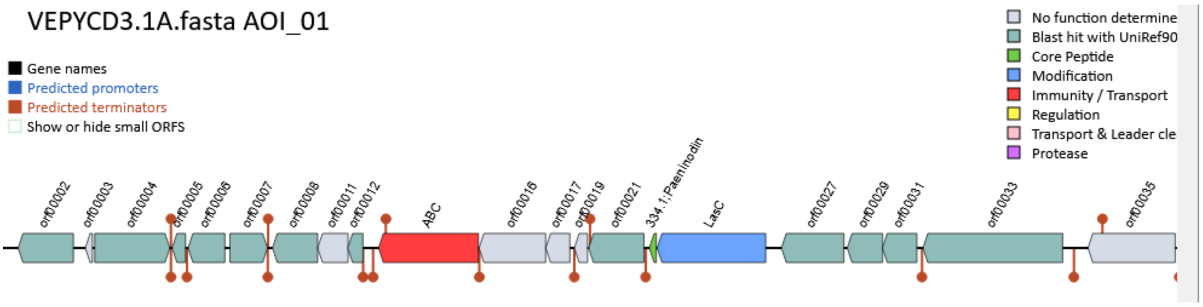

Suppl. Fig. S: Paeninodin (334.1) / LasC/ABC gene cluster in Bacillus sp. CD3.1A VEPY 1 and B. pacificus HD1,3

```
Query        MKKDWTIPTLEVLDINMTMAGPGLKTPDAVQPDIDEVVHY
                                     AGPG  TPDA QPD DE VHY
Paeninodin                           AGPGTSTPDAFQPDPDEDVHYDS
```

Subclass Lasso peptide

Organism Paenibacillus dendritiformis C454

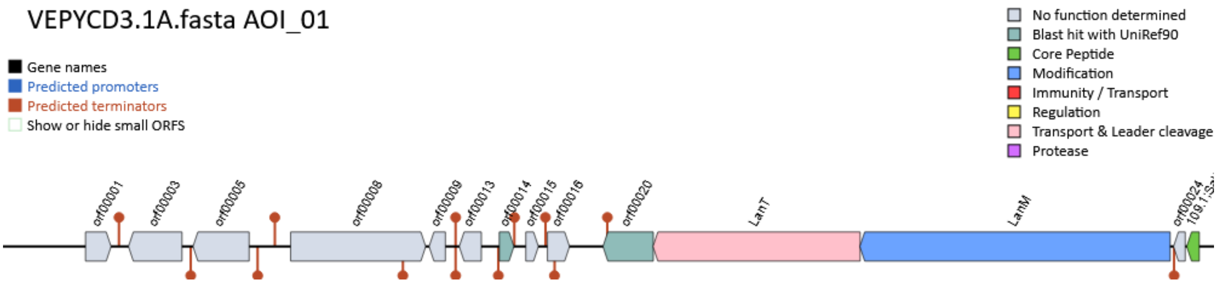

Salivaricin A (109.1)/LanT/ LanM gene cluster in Bacillus sp. CD3.1A VEPY 5.1

```
Query                   MEELKSVVMTVTDEELQE-AAGAAGCGWLCTVTDDCPNSVFVCC
                           L +V+  V+++EL E A G  G GW  T+TDDCPNSVFVCC
SalivaricinA MERRMSFMKNSKDILTNVIEEVSEKELMEVAGGKKGSGWFATITDDCPNSVFVCC
```

Subclass Lanthipeptide B

Organism Streptococcus pyogenes MGAS10394

Suppl. Figure S8. RiPP gene clusters detected by applying the BAGEL4 software (http://bagel4.molgenrug.nl/) in the Vietnamese *Bacillus cereus* group genomes.

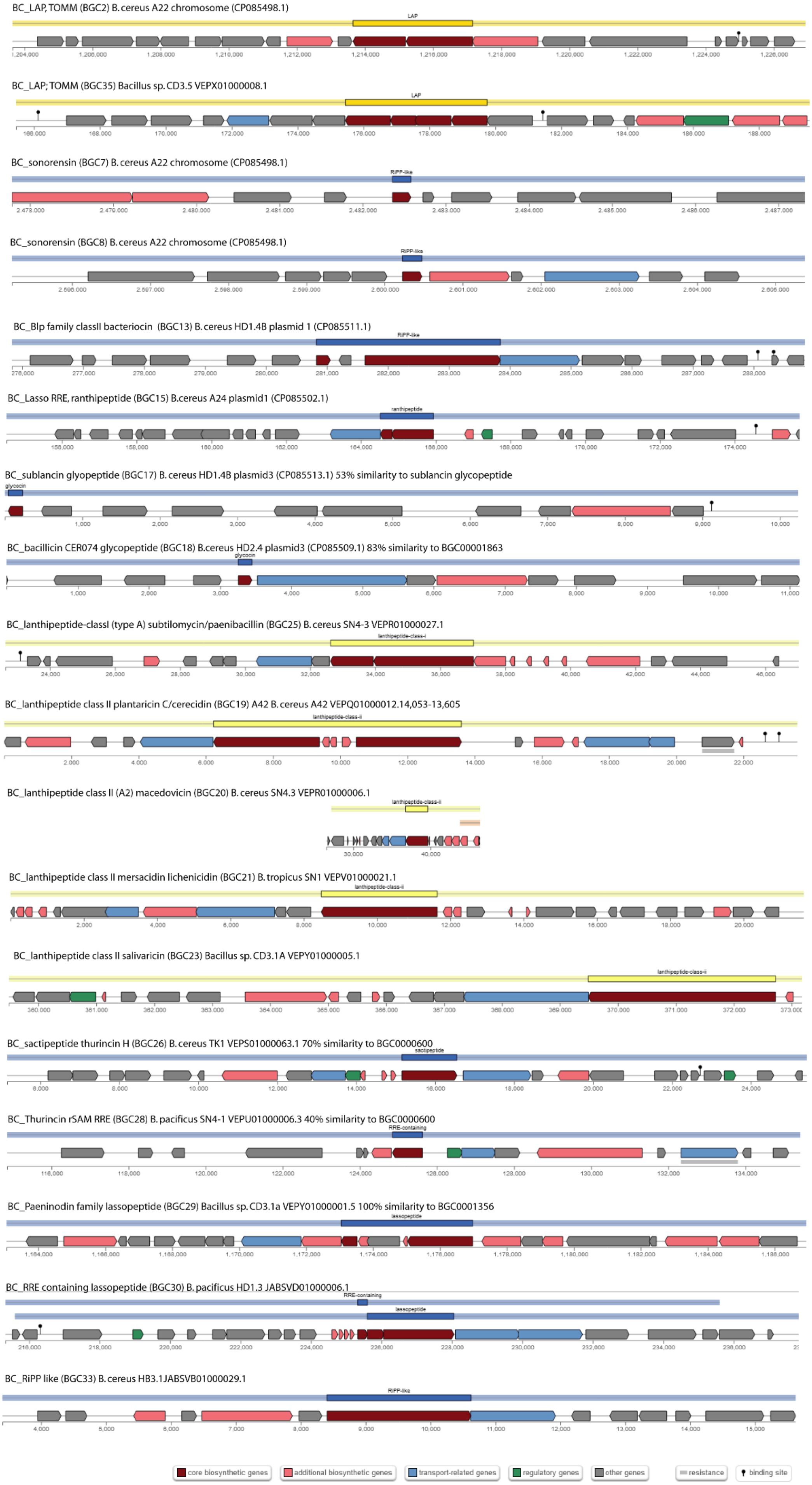
BC_LAP, TOMM (BGC2) B. cereus A22 chromosome (CP085498.1)
BC_LAP; TOMM (BGC35) Bacillus sp. CD3.5 VEPX01000008.1
BC_sonorensin (BGC7) B. cereus A22 chromosome (CP085498.1)
BC_sonorensin (BGC8) B. cereus A22 chromosome (CP085498.1)
BC_Blp family classII bacteriocin (BGC13) B. cereus HD1.4B plasmid 1 (CP085511.1)
BC_Lasso RRE, ranthipeptide (BGC15) B.cereus A24 plasmid1 (CP085502.1)
BC_sublancin glyopeptide (BGC17) B. cereus HD1.4B plasmid3 (CP085513.1) 53% similarity to sublancin glycopeptide
BC_bacillicin CER074 glycopeptide (BGC18) B.cereus HD2.4 plasmid3 (CP085509.1) 83% similarity to BGC00001863
BC_lanthipeptide-classI (type A) subtilomycin/paenibacillin (BGC25) B. cereus SN4-3 VEPR01000027.1
BC_lanthipeptide class II plantaricin C/cerecidin (BGC19) A42 B. cereus A42 VEPQ01000012.14,053-13,605
BC_lanthipeptide class II (A2) macedovicin (BGC20) B. cereus SN4.3 VEPR01000006.1
BC_lanthipeptide class II mersacidin lichenicidin (BGC21) B. tropicus SN1 VEPV01000021.1
BC_lanthipeptide class II salivaricin (BGC23) Bacillus sp. CD3.1A VEPY01000005.1
BC_sactipeptide thurincin H (BGC26) B. cereus TK1 VEPS01000063.1 70% similarity to BGC0000600
BC_Thurincin rSAM RRE (BGC28) B. pacificus SN4-1 VEPU01000006.3 40% similarity to BGC0000600
BC_Paeninodin family lassopeptide (BGC29) Bacillus sp. CD3.1a VEPY01000001.5 100% similarity to BGC0001356
BC_RRE containing lassopeptide (BGC30) B. pacificus HD1.3 JABSVD01000006.1
BC_RiPP like (BGC33) B. cereus HB3.1JABSVB01000029.1
core biosynthetic genes
additional biosynthetic genes
transport-related genes
regulatory genes
other genes
resistance
binding site

Suppl. Figure S9: RiPP gene clusters detected by applying the antiSMASH version 6 software in the Vietnamese *Bacillus cereus* group genomes.
