# Supplementary material for: Plant-Associated Representatives of the Bacillus cereus Group Are a Rich Source of Antimicrobial Compounds
Source: Microorganisms. 2023 Oct 31;11(11):2677. doi: 10.3390/microorganisms11112677 (PMC10672896; doi:10.3390/microorganisms11112677)
Supplement: Supplementary file 1 [file Suppl. Fig.S3.pdf › Suppl. Fig.S3.pdf]

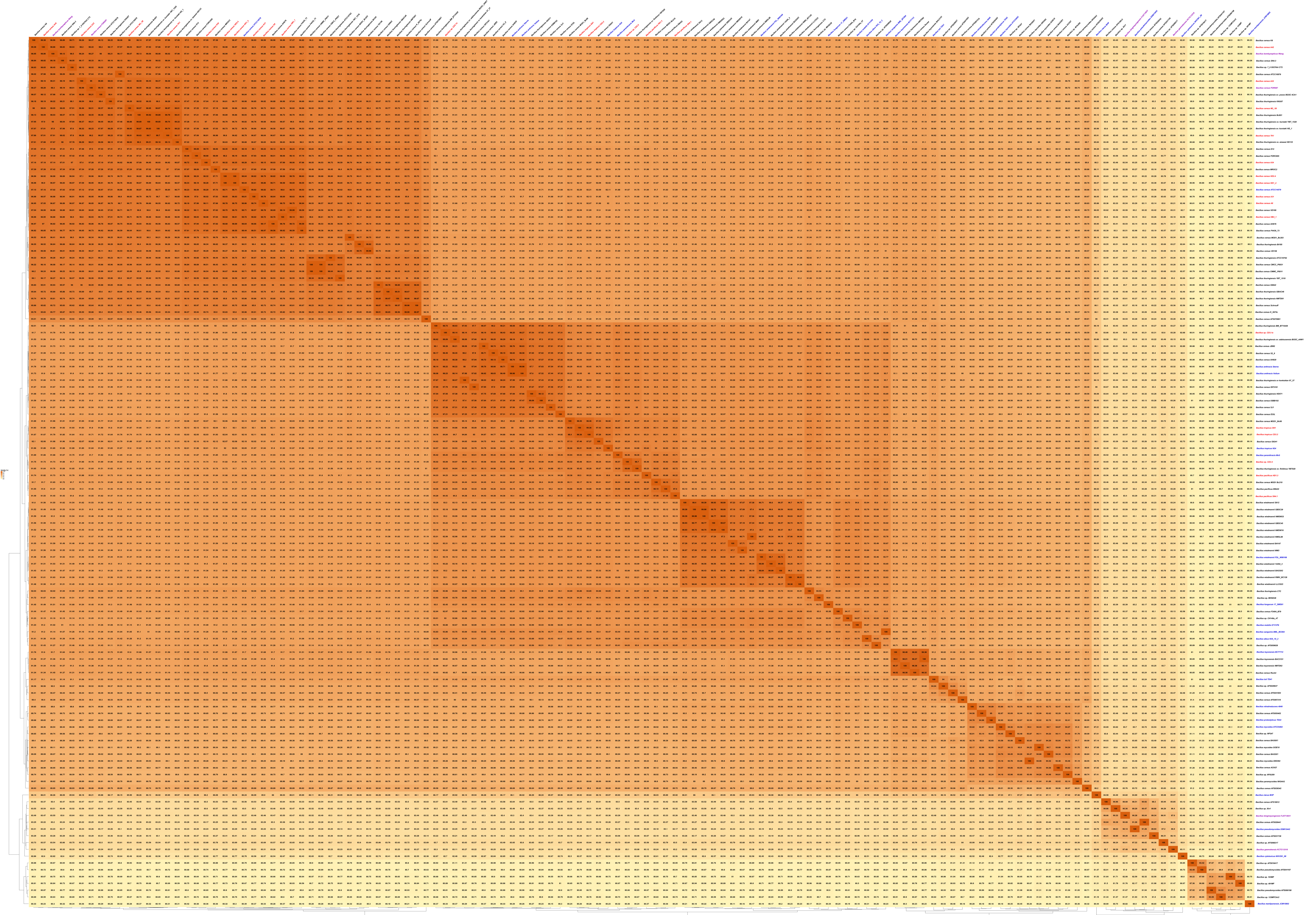

Suppl. Fig. S3: Heat map of the FastANI values of 128 B. cereus group genomes calculated and drawn by the EDGAR software package. The species cutoff value is <96%, the subspecies cutoff is <98%. The figure corresponds to Figure 1A and uses the same labelling for the Vietnamese isolates (red) and the type strains (blue).
